# Interferon *α* Enhances B Cell Activation Associated With FOXM1 Induction: Potential Novel Therapeutic Strategy for Targeting the Plasmablasts of Systemic Lupus Erythematosus

**DOI:** 10.3389/fimmu.2020.498703

**Published:** 2021-02-03

**Authors:** Kanae Akita, Ken Yasaka, Tsuyoshi Shirai, Tomonori Ishii, Hideo Harigae, Hiroshi Fujii

**Affiliations:** ^1^Department of Hematology and Rheumatology, Tohoku University Graduate School of Medicine, Sendai, Japan; ^2^Department of Clinical Research, Innovation and Education Center, Tohoku University Hospital, Sendai, Japan

**Keywords:** systemic lupus erythematosus, B cell, interferon *α*, plasmablast, FOXM1

## Abstract

Systemic lupus erythematosus (SLE) is an autoimmune disease. It is characterized by the production of various pathogenic autoantibodies and is suggested to be triggered by increased type I interferon (IFN) signature. Previous studies have identified increased plasmablasts in the peripheral blood of SLE patients. The biological characteristics of SLE plasmablasts remain unknown, and few treatments that target SLE plasmablasts have been applied despite the unique cellular properties of plasmablasts compared with other B cell subsets and plasma cells. We conducted microarray analysis of naïve and memory B cells and plasmablasts (CD38^+^CD43^+^ B cells) that were freshly isolated from healthy controls and active SLE (n = 4, each) to clarify the unique biological properties of SLE plasmablasts. The results revealed that all B cell subsets of SLE expressed more type I IFN-stimulated genes. In addition, SLE plasmablasts upregulated the expression of cell cycle-related genes associated with higher FOXM1 and FOXM1-regulated gene expression levels than that in healthy controls. This suggests that a causative relationship exists between type I IFN priming and enhanced proliferative capacity through FOXM1. The effects of pretreatment of IFN*α* on B cell activation and FOXM1 inhibitor FDI-6 on B cell proliferation and survival were investigated. Pretreatment with IFN*α* promoted B cell activation after stimulation with anti-IgG/IgM antibody. Flow cytometry revealed that pretreatment with IFN*α* preferentially enhanced the Atk and p38 pathways after triggering B cell receptors. FDI-6 inhibited cell division and induced apoptosis in activated B cells. These effects were pronounced in activated B cells pretreated with interferon *α*. This study can provide better understanding of the pathogenic mechanism of interferon-stimulated genes on SLE B cells and an insight into the development of novel therapeutic strategies.

## Introduction

In systemic lupus erythematosus (SLE), abnormal B cells and T cells trigger the production of autoantibodies, such as anti-dsDNA antibody, forming immune complexes and damaging the tissues (1). Previously, it is reported that PBMC of SLE patients express more interferon-stimulated genes (ISGs) (2, 3) and type I IFN enhance B cell differentiation (4). The production of type I IFN from plasmacytoid dendritic cells is considered to contribute to SLE pathogenesis (5).

The main treatment strategy for SLE is the use of steroids and immunosuppressants to prevent the production of autoantibodies (6). In addition to conventional therapies, belimumab targeting BAFF that regulates B cell activation and survival has been used in clinical practice (7). Moreover, novel biologics and synthetic molecules have been investigated for SLE therapy (8). However, among these therapies, the double-blind randomized control studies of rituximab targeting B cells (9) and bortezomib targeting plasma cells did not exhibit any significant effect on SLE activity without severe side effects (10, 11), despite the efficacy in non-randomized studies or case series studies (12).

In terms of the SLE pathogenesis, IFN*α*-targeting therapy was a promising strategy for the treatment of SLE. Recently, the phase III clinical trial of anifrolumab for SLE patients exhibited a significant effect on steroid reduction and skin lesions (13, 14); however, it still remains a question as to what kind of patients will benefit from type I IFN-targeting therapies (15). SLE treatment still have unmet needs and require further understanding of its pathogenesis involving type I IFN.

Plasmablasts defined as CD27^hi^CD38^+^ are considered to be plasma cell precursors (16). Since plasmablasts in peripheral blood are dividing, migratory, and antibody-producing (17), this cell population exhibits unique cellular properties compared with other B cell subsets (*e.g.*, naïve and memory B cells) and plasma cells. It can also be an attractive therapeutic target in SLE treatment. Recently, single-cell RNA sequencing revealed that several B cell subclusters and a plasma cell subcluster with ISGs were identified in SLE patients (18); however, the unique biological properties or molecular targets of plasmablasts in SLE are yet to be determined. First, we characterized CD19^+^CD38^+^CD43+ B cell population as a phenotype of plasmablasts which secrete immunoglobulins. Next, we conducted microarray analysis by comparing freshly isolated naïve B cells, memory B cells, and CD38^+^CD43^+^ B cells, which are considered to be plasmablasts, of healthy donors and active SLE patients to investigate the SLE plasmablast-specific activated pathway. We found that cell cycle signature in SLE plasmablasts were significantly increased, as well as type I IFN signatures in naïve and memory B cells, and plasmablasts. Additionally, transcriptional factor, FOXM1 and its downstream molecules were significantly increased in SLE plasmablasts. FOXM1 is a master regulator for cell proliferation and survival (19), and its inhibitors have therapeutical effect on tumors (20, 21). We hypothesized that a causal relationship exists between type I IFN signature and cell cycle signature, and FOXM1 may play a critical role in cell proliferation of activated B cells. In the present study, we investigated the effect of pretreatment with IFN*α* on B cell activation and the effect of FOXM1 inhibitor on B cell proliferation and survival.

## Methods

### Patients and Healthy Controls

Patients diagnosed with SLE according to the 1997 American College of Rheumatology classification criteria (22) were recruited at Tohoku University Hospital, Japan. The disease activities of SLE were assessed using the SLEDAI scoring system. Healthy donors were recruited from the Department of Hematology and Rheumatology, Tohoku University Graduate School of Medicine, Japan. The present study was approved by the Medical Ethical Review Board at Tohoku University School of Medicine. The donor profiles of SLE patients used in the clinical correlation (SLE1–SLE8) and microarray analysis (SLE9–SLE12) were presented in **Supplementary Tables 1** and **Table 1**, respectively.

**Table 1:** Top 10 of upstream analysis associated with DEGs of CD38^+^CD43^+^ B cells between healthy donors and SLE.

Symbol	Entrez Gene Name	*Fold Change	-log_10_(p-value)	z-score
TBX2	T-Box Transcription Factor 2	1.644	22.21	5.161
E2F3	E2F Transcription Factor 3	1.327	17.69	4.801
E2F1	E2F Transcription Factor 1	3.332	17.52	3.907
E2F2	E2F Transcription Factor 2	2.606	14.99	2.364
MITF	Microphthalmia-associated transcription factor	1.223	11.63	5.588
FOXM1	Forkhead box protein M1	4.139	11.04	5.483
MYOD1	Myogenic Differentiation 1	10.56	8.92	2.455
STAT1	signal transducer and activator of transcription 1	4.761	7.59	3.953
IRF7	interferon regulatory factor 7	2.734	7.06	4.314
IRF1	interferon regulatory factor 1	-1.164	5.98	3.743

*Fold Change; CD38^+^CD43^+^ of SLE vs CD38^+^CD43^+^ of HC.

### Flow Cytometry

The following antibodies were utilized for cell surface staining (fluorescence-labeled monoclonal antibodies against human were obtained from BD Biosciences, unless otherwise stated): PE anti-CD43, APC anti-CD38, V500 anti-CD27, PerCP-Cy5.5 anti-CD19, V450 anti-CD3, V450 anti-CD14, and V450 anti-CD16 (all from BD Biosciences). The following antibodies were utilized for intracellular staining: Zenon Alexa Fluor 488 Rabbit IgG Labeling Kit (Invitrogen), rabbit IgG isotype control (Cell Signaling Technology, Danvers, MA). For phosphoprotein analysis, resting or stimulated B cells were fixed with BD Phosflow Fix Buffer I (BD Bioscience) and permeabilized with Perm Buffer III (BD Bioscience). Subsequently, the B cells were stained with Violet Fluorescent Cell Barcoding Dye 450 and 500 (BD Bioscience) to be distinguished by different stimuli and time points. After washing, cells were stained with the following Alexa Fluor 647-conjugated antibodies: anti-PTEN (Phosphatase and Tensin Homolog Deleted from Chromosome 10), anti-Syk (Spleen tyrosine kinase) (pY352), anti-Btk (Bruton’s tyrosine kinase) (pY223), anti-ERK (extracellular signal-regulated kinase)1/2 (pT202/pY204), anti-p38 (pT180/pY182), anti-NF-*κ*B (nuclear factor-kappa B) p65 (pS529), anti-I*κ*B*α* (NF-*κ*B inhibitor *α*), anti-Akt (protein kinase B) (pS473), anti-mTOR (mammalian target of rapamycin) (pS2448), anti-S6 (ribosomal S6 kinase) (pS235/pS236), mouse IgG1 isotype, and mouse IgG2b isotype (all from BD Biosciences). For the detection of apoptotic cells, Near SR LIVE/DEAD fixable dead cell stains (Thermo Fisher Scientific) and APC-Annexin V (BD Biosciences) were used. Flow cytometry was performed using the FACSCanto II system (BD Biosciences), and data were analyzed using the FlowJo Software (BD Biosciences) and the Cytobank platform (Cytobank Inc.).

### B Cell Isolation

Peripheral blood mononuclear cells (PBMCs) were freshly isolated from healthy donors or SLE patients *via* Ficoll (GE Healthcare, Uppsala, Sweden) gradient centrifugation. For the microarray analysis, first, B cells were positively isolated with CD19 microbeads (Miltenyi Biotec, Auburn, CA), then CD19^+^CD27^−^ CD38^−^CD43^−^CD3^−^CD14^−^CD16^−^(naïve B cell), CD19^+^CD27^+^ CD38^−^CD43^−^CD3^−^CD14^−^CD16^−^ (memory B cell), and CD19^+^CD38^+^CD43^+^CD3^−^CD14^−^CD16^−^ (CD38^+^CD43^+^ B cell) were gated and isolated using FACSAria II (BD). For B cell cultures, B cells were isolated from PBMCs using the B Cell Isolation Kit II (Miltenyi Biotec).

### B Cell Culture

For culture medium preparation, we used RPMI 1640 (Sigma–Aldrich, Saint Louis, MI) supplemented with 10% (v/v) heat-inactivated fetal bovine serum (FBS) (Biological Industries, Cromwell, CT), 100 U/ml penicillin, and 100 µg/ml streptomycin. F (ab′)_2_ Fragment Goat Anti-human IgG + IgM (H + L) (Jackson ImmunoResearch, West Grove, PA) was added at a final concentration of 50 µg/ml. Human IFN*α* A/D (Bg/II) was obtained from PBL Assay Science (Piscataway, NJ), and MEGACD40L was purchased from Enzo Life Science (NY) and added to the culture to stimulate CD40. The FOXM1 inhibitor FDI-6 was purchased from Axon Medchem (Groningen, Netherlands). Freshly isolated B cells were incubated in 96-well U-bottom plates (Falcon, Durham, NC) at a concentration of 1 × 10^5^ cells/200 µl. B cells were stimulated with anti-IgG/IgM (50 µg/ml) 6 h after B cell isolation. For pretreatment with IFN*α* (pre-IFN), 1,000 U/ml of IFN*α* was added 6 h before and during anti-IgG/IgM stimulation. In some experiments, 100 ng/ml MEGACD40L (Enzo Life Sciences) was simultaneously added with stimulation with anti-IgG/IgM. All culture experiments were conducted in triplicate.

### ELISPOT Assay

IgG secretion was determined by ELISPOT assay using Human IgG ELISpotBASIC kit (Mabtech, Cincinnati, OH). Mouse anti-human IgG antibodies (1.5 ug/ml) were coated on the PVDF-membrane in MultiScreen-IP plates (Merck Millipore, Carrigtwohill, Ireland) over night. After washing, the membranes were blocked with PBS containing 1% BSA. Subsequently, CD38^−^CD43^−^ B cells and CD38^+^CD43^+^ B cells isolated with flow cytometry were seeded onto each well with the cell number of 500, 100, 20/well. After 24 h incubation in RPMI1640 supplemented with 10% FBS, the membranes were washed, and biotinylated anti-IgG Fc antibodies (2 ug/ml) were added. After washing, the membranes were incubated with streptavidin-horseradish oxidase and developed with substrate for ELISpot (Mabtech).

### Microarray Analysis of Isolated B Cell Subsets

Microarray data were collected from freshly isolated naïve B cells, memory B cells, and CD38^+^CD43^+^ B cells of four SLE patients and four healthy donors. After the isolation of each B cell subset, the total RNA was extracted in each B cell subset using TRIzol (Invitrogen, Carlsbad, CA) and RNeasy Mini Kit (QIAGEN, Hilden, Germany). Briefly, cell pellets were lysed in TRIzol and chloroform was added. After centrifugation, upper layers were mixed with equal volume of ethanol, and the mixtures were transferred to spin columns of RNAeasy Mini Kit. After washing the columns, total RNAs were eluted with RNAse free water. After amplification of targeted cRNA and labeling with Cyanine-3 using Low Input Quick Amp Labeling Kit (Agilent Technologies, Santa Clara, CA), fragmented cRNAs were applied to Whole Human Genome Oligo microarray 8 × 60 K v2 (Agilent Technologies) and then hybridized. The array slides were scanned using Agilent’s Microarray Scanner System (Agilent Technologies). The obtained microarray data were normalized using the GeneSpring 12.5 software (Agilent Technologies). Differentially expressed genes (DEGs) were determined by comparing the gene expression levels of each B cell subset (naïve, memory, and CD38^+^CD43^+^CD27^+^ B cells) between SLE patients and healthy donors. DEGs in each comparison were defined as significantly changed (p < 0.05, moderate *t*-test) and more than two-fold upregulated or less than 0.5-fold downregulated genes. Heat maps were generated after hierarchical clustering. Canonical pathway and upstream analyses of DEGs were conducted using the Ingenuity Pathway Analysis program (QIAGEN). Significantly activated pathways and upstream molecules (transcriptional factors) were defined as significantly different (p < 0.01) using Fisher’s exact test and a Z score > 2.0. Type I IFN signature genes (23 genes) (23) and cell cycle signature genes (231 genes) (24) were calculated as follows: first, the maximum value in any gene row was normalized to be 1. Then, the sum of the value of each sample was obtained to calculate the gene expression scores. To identify genes that specifically changed in the SLE plasmablast, similar entity analysis was conducted using the GeneSpring software with cutoff range of 0.6–1.0 using the DEGs of the plasmablast. The microarray data were deposited in Gene Expression Omnibus [GSE156751].

### Cell Division

Isolated B cells were labeled using CellTrace Violet Cell Proliferation Kit (Invitrogen) and analyzed *via* flow cytometry after the culture was prepared. Cell proliferation was assessed using a proliferation index (PI). PI denotes the average number of cell divisions and is calculated using the following formula. $\text{PI}=\frac{\Sigma_{0}^{i}N_{i}}{\Sigma_{0}^{i}N_{i}/2^{i}}$

where *N* denotes the percentage of B cells in each generation, and *i* denotes the number of cell divisions.

### Quantitative Real-Time PCR

After the total RNA was extracted from B cells using the RNeasy Mini Kit, cDNA was synthesized using the ReverTra Ace qPCR RT Kit (TOYOBO, Osaka, Japan). The following primers were used: 18S ribosomal RNA forward primer : 5′-AGGAATTCCCAGTAAGTGCG-3′, 18S ribosomal RNA reverse primer : 5′-GCCTCACTAAACCATCCAA-3′, FOXM1 forward primer : 5′-ATACGTGGATTGAGGACCACT-3′, FOXM1 reverse primer : 5′-TCCAATGTCAAGTAGCGGTTG-3′, CCNA2 forward primer : 5′-CGCTGGCGGTACTGAAGTC-3′, and CCNA2 reverse primer : 5′-GAGGAACGGTGACATGCTCAT-3′.

Quantitative real-time PCR (qPCR) was performed using the QuantiTect SYBR Green PCR Kit (QIAGEN) with the C1000 Thermal Cycler (Bio-Rad, Hercules, CA). The calculated copy numbers were normalized according to the 18S rRNA expression. The gene expression levels were standardized by each corresponding 18S rRNA expression level.

### Western Blotting

After 120 min stimulation of B cells isolated from healthy donors by anti-IgG/IgM antibody with or without pre-IFN treatment, cells were lysed with 1x SDS sample buffer (Cell Signaling Technologies) and sonicated. The cell lysates were electrophoresed on 12% polyacrylamide gel and then transferred onto Immobilon transfer membranes (Millipore). The membranes were treated with rabbit anti-phosphorylated Akt (Cell Signaling Technologies), rabbit anti-phoshorylated p38 (Cell Signaling Technologies), and rabbit anti-tubulin antibody (abcam, Cambridge, UK). IRDye 800CW-conjugated goat anti-rabbit (LI-COR Biosciences, Lincoln, NE) was used as a secondary antibody. Fluorescence intensity was measured, and densitometric analysis was performed with an Odyssey Infrared Imaging System (LI-COR Biosciences).

### Statistical Analysis

Data are expressed as mean ± SD. The statistical significance of the differences was determined *via* ANOVA, paired and unpaired *t*-test. Correlation coefficients and statistical significance between the frequencies of CD38^+^CD43^+^ B cells and SLEDAI scores were obtained with Spearman’s rank correlation analysis. Moreover, the differences were considered statistically significant for *p*-values less than 0.05. Statistical analyses were conducted using the GraphPad Prism 7 software (San Diego, CA).

## Results

### CD38^+^CD43^+^ B Cells Have High Validity as Plasmablasts

**Figure 1:**
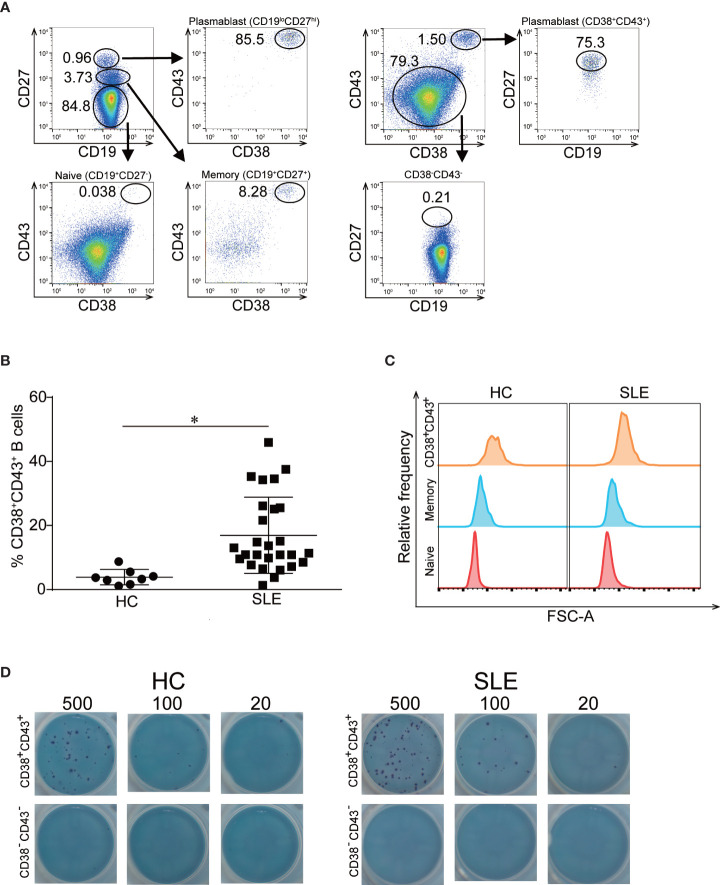
Validation of CD38^+^CD43^+^ B cells as plasmablasts. **(A)** Consistency in B cell surface markers. PBMCs from a healthy control (HC) were labeled with fluochrome-tagged antibodies to CD3, CD14, CD16, CD19, CD27, CD38, and CD43. After gating CD3^−^CD14^−^CD16^−^ cells, CD19^lo^CD27^hi^ B cells or CD38^+^CD43^+^ B cells were gated and the consistency of these two subsets were analyzed. **(B)** Percentage of CD38^+^CD43^+^ cells among CD19^+^ B cells in HC and active SLE patients. PBMCs from HC (n = 8) and active SLE with SLEDAI ≥6 (n = 27) were isolated and evaluated the percentage of CD38^+^CD43^+^ B cells in CD19 gated cells. The error bars represent SD. Statistical analysis was performed with unpaired *t*-test. *p < 0.01. **(C)** Cell size of B cell subsets. The intensity value of FSC-A is displayed as histograms in naïve B cells, memory B cells and CD38^+^CD43^+^ B cells from a healthy donor (left) and a SLE patient (right). Data in **(A, C)** are representative data from three independent donors, each. **(D)** ELISPOT analysis evaluating antibody secreting capacity of isolated B cells. CD38^+^CD43^+^ B cells and CD38^−^CD43^−^ B cells were isolated with flowcytometry. Each B cell subset with cell number indicated was incubated on membranes coated with anti-human IgG antibody in RPMI1640 containing 10% FBS. After 24 h incubation and washing, spots were developed.

Conventionally, plasmablasts are considered as CD19^lo^CD27^hi^CD38^hi^ B cells (25). In flow cytometric analysis, CD19^lo^CD27^hi^CD38^hi^ B cells were presumably overlapped with the population of CD19^+^CD27^+^ memory B cells and are difficult to be precisely gated or sorted from other cells. It has been reported that CD20^+^CD27^+^CD43^+^ B cells exhibit similar characteristics to those of plasmablasts (26). We compared CD38^+^CD43^+^ with CD19^lo^CD27^hi^ B cells *via* flow cytometry and found that CD38^+^CD43^+^ B cells were highly overlapped with CD19^lo^CD27^hi^ B cells (**Figure 1A**). The frequencies of CD38^+^CD43^+^ cells among CD19^+^ B cells were significantly elevated in active SLE than healthy controls (**Figure 1B**) and significantly reduced after intensification of the immunosuppressive therapy for active SLE and correlated with SLEDAI scores (**Supplementary Figure 1**). To determine whether CD38^+^CD43^+^ B cells are eligible for plasmablasts, we evaluated the cell size, gene expression, and antibody secreting capacity of the B cell subset. The cell size was prominently increased in CD38^+^CD43^+^ B cells of both healthy donors and SLE patients (**Figure 1C**). The expressions of IRF4 and PRDM1 were increased in plasmablasts (27) and, as revealed by microarray analysis, were significantly increased in CD38^+^CD43^+^ B cells of both healthy donors and SLE patients (**Supplementary Figures 2A and B**). Isolated CD38^+^CD43^+^ B cells were shown to have the capacity to secrete IgG, whereas CD38^−^CD43^−^ B cell not (**Figure 1D**). Additionally, CD38^+^CD43^+^ B cells can be clearly gated from other cells (**Supplementary Figure 3**). We considered CD38^+^CD43^+^ B cells as plasmablasts and gated and sorted CD38^+^CD43^+^ B cells for the analysis of plasmablasts in the following experiments.

### The Gene Expressions of Type I IFN and Cell Cycle Signatures Are Upregulated, and Increased Expression of the Transcription Factor FOXM1 Is Identified in CD38^+^CD43^+^ B Cells of SLE Patients

**Figure 2:**
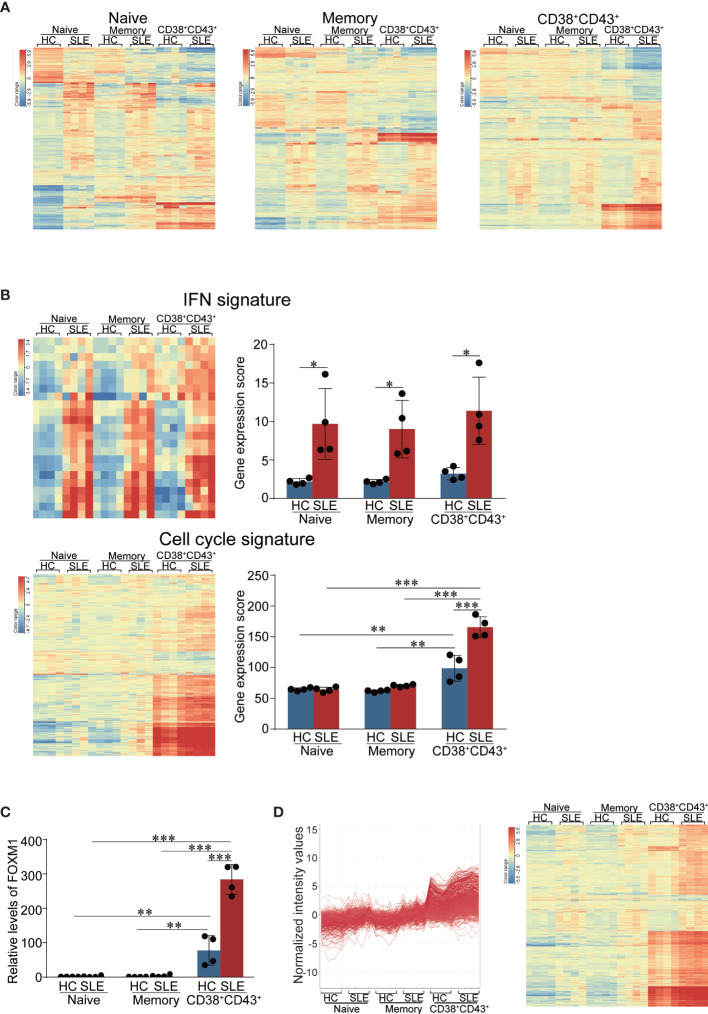
The correlation of DEGs in CD38^+^CD43^+^ B cells of SLE with the canonical pathways related to cell cycle as well as type I IFN. **(A)** DEGs of SLE patients compared with healthy donors in each B cell subset. The heatmap represents the DEGs of each B cell subset (naïve, memory, and CD38^+^CD43^+^ B cells) of SLE patients compared with healthy donors. Significantly upregulated (>twofold) and downregulated (<0.5-fold) genes were shown after hierarchical clustering analysis. Statistical analysis was conducted *via* moderate *t*-test using the GeneSpring software. **(B)** The gene expression score of type I IFN signature and cell cycle signature in each B cell subset. IFN signature genes (23 genes) (23) (upper) and cell cycle signature genes (231 genes) (24) (lower) were selected as representative genes for each pathway. The heatmap represents the gene expression levels of type I IFN signatures and cell cycle signatures in each B cell subset (left). After the expression levels of IFN signature were normalized to be 1 for the maximum value in any gene row, the sum of the values of each B cell subset in healthy donors and SLE patients was obtained to calculate the gene expression levels and scored (right). The error bars represent SD. **(C)** FOXM1 gene expression level in each B cell subset. FOXM1 expression was assessed in naïve, memory, and CD38+CD43+ B cells between healthy donors and SLE patients. Gene expression was normalized to be 1 for the minimum value in any B cell subset. The error bars represent SD. **(B, C)** Statistical analysis was conducted *via* one-way ANOVA using Tukey’s multiple comparison test.*p < 0.05, **p < 0.01, and ***p < 0.001. Data were obtained from four independent healthy donors and four independent active SLE patients. **(D)** Genes exhibiting similar expression patterns to FOXM1. Among DEGs in CD38^+^CD43^+^ B cells, “find similar entities” analysis with a correlation cut-off range of 0.6 ≤ r ≤ 1.0 resulted in the selection of 725 genes. Histogram (left) and heatmap (right) show the gene expression levels in each B cell subset.

To determine DEGs between healthy donors and SLE patients in naïve, memory, and CD38^+^CD43^+^ B cells, we compared the gene expression profiles of each B cell subset of SLE patients with those of healthy donors. The purity of the isolated cells was comparable between healthy donors and SLE patients (**Supplementary Figure 3**). **Figure 2A** presents the DEGs in each B cell subset. A total of 594 genes were upregulated, and 595 genes were downregulated in the naïve B cell subset; 527 genes were upregulated, and 381 genes were downregulated in the memory B cell subset; and 1,452 genes were upregulated, and 723 genes were downregulated in the CD38^+^CD43^+^ B cell subset. The top 30 upregulated or downregulated genes among the DEGs are presented in **Supplementary Table 3**.

Canonical pathway analysis on IPA revealed that several cell-cycle-related pathways were activated, as well as interferon signaling, whereas only the interferon signaling pathway was significantly activated in each of the naïve and memory B cell subset (**Table 2**). We calculated the gene expression scores (**Figure 2B**) after the selection of type I IFN signature genes (23 genes) (23) and cell cycle signature genes (231 genes) (24) from the microarray data of healthy donors and SLE patients. IFN signature gene expressions were significantly increased in each B cell subset of SLE patients compared with healthy donors (**Figure 2B**, upper). Moreover, the gene expressions of cell cycle signature were higher only in CD38^+^CD43^+^ B cells of SLE patients than in those of healthy donors (**Figure 2B**, lower). Upstream analysis revealed that several transcriptional factors related to cell cycle, as well as IFN-related factors, were significantly upregulated in CD38^+^CD43^+^ B cells of SLE patients more than in those of healthy donors (**Table 1**). Among the activated transcriptional factors, the expressions of E2F1, E2F2, and FOXM1 genes were shown to be more significantly upregulated in CD38^+^CD43^+^ B cells of SLE patients compared with other B cell subsets (**Figure 2C** and **Supplementary Figure 4**). We consider the gene expression pattern of FOXM1, which is a master regulator for cell cycle and cell survival (19), as a prototype of SLE plasmablast-specific genes; hence, we conducted an analysis of find similar entities for FOXM1 from DEGs in the CD38^+^CD43^+^ B cell subset to determine the genes that are significantly/uniquely upregulated in CD38^+^CD43^+^ B cells of SLE patients. Furthermore, 725 genes were chosen as similar entities to FOXM1 (**Figure 2D**). Canonical pathway analysis revealed that the genes of entities similar to FOXM1 strongly correlated with the cell cycle (**Table 3**).

**Table 2:** Canonical pathways associated with DEGs between healthy donors and SLE.

Ingenuity Canonical Pathways	−log_10_ (p-value)	z-score
**Naïve B cell**		
Interferon Signaling	5.68	3
**Memory B cell**		
Interferon Signaling	8.29	3.464
**CD38^+^CD43^+^ B cell**		
Kinetochore Metaphase Signaling Pathway	12	3.024
Cell Cycle Control of Chromosomal Replication	8.81	4.264
Estrogen-mediated S-phase Entry	5.07	2.714
Cyclins and Cell Cycle Regulation	4.01	2.84
Interferon Signaling	3.02	3.162
RAN Signaling	2.66	2.449

**Table 3:** Canonical pathways associated with the genes of the similar entities to FOXM1.

Ingenuity Canonical Pathways	−log_10_(p-value)	z-score
Kinetochore Metaphase Signaling Pathway	22.9	2.887
Cell Cycle Control of Chromosomal Replication	14.8	4.472
Estrogen-mediated S-phase Entry	8.04	2.53
Cyclins and Cell Cycle Regulation	7.79	2.673
Pyrimidine Deoxyribonucleotides *De Novo* Biosynthesis I	4.18	2.449
RAN Signaling	3.76	2.236
Cell Cycle Regulation by BTG Family Proteins	3.56	2.449
Aryl Hydrocarbon Receptor Signaling	2.46	2.111

### Pretreatment With IFN*α* Promoted Cell Enlargement, Cell Division, and Gene Expression in B Cells Stimulated *via* BCR

**Figure 3:**
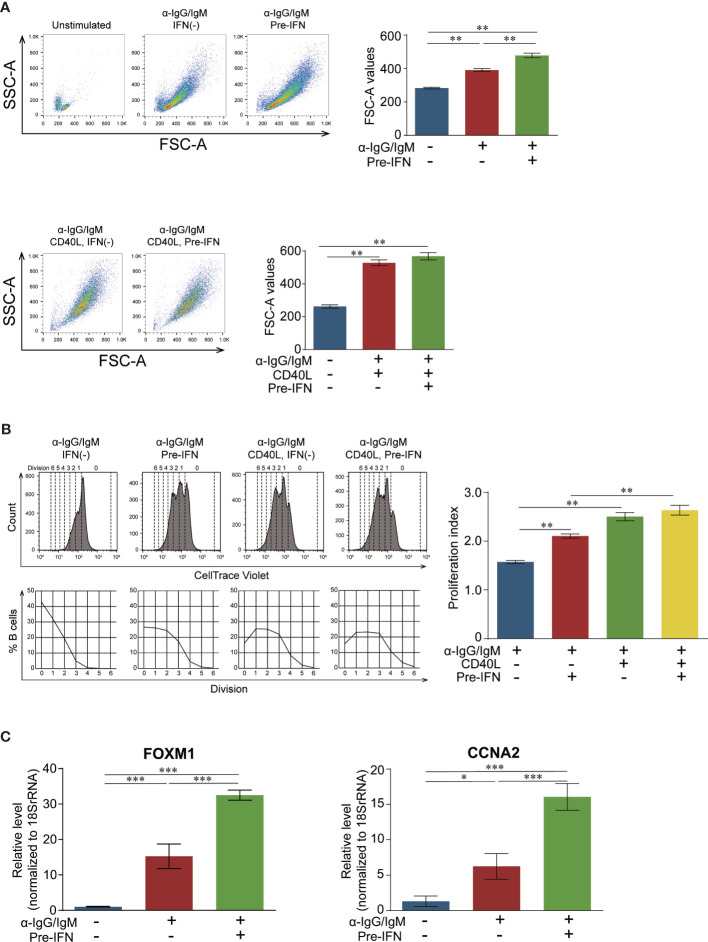
Enhanced B cell stimulation with pre-IFN. **(A)** Effects of pre-IFN on B cell enlargement after stimulation with anti-IgG/IgM antibody and CD40L. Isolated B cells from a healthy donor were stimulated with *α*-IgG/IgM (50 µg/ml) with or without IFN*α* (1,000 U/ml) or CD40L (100 ng/ml) pretreatment. Cell size was measured through FSC-A intensity and analyzed 3 d after the start of the culture. The geometric mean of FSC-A is presented in each cultured B cell. **(B)** The effects of pre-IFN on B cell division after stimulation with anti-IgG/IgM antibody and CD40L. Peripheral B cells from a healthy donor were labeled using CellTrace Violet Cell Proliferation Kit and stimulated with *α*-IgG/IgM (50 µg/ml) with or without pre-IFN (1,000 U/ml) or CD40L (100 ng/ml). Cell division was analyzed *via* flow cytometry 6 d after the beginning of the culture. Left: Cell division is presented as histograms, and the number of cell divisions is indicated above the histograms (upper). B cell percentages in each generation are plotted on a chart (lower). Right: Proliferation indices (PI) were calculated in each stimulated B cell. **(C)** The effects of pre-IFN on FOXM1 and CCNA2 gene induction after stimulation with anti-IgG/IgM antibody. Isolated B cells from a healthy donor were stimulated with *α*-IgG/IgM (50 µg/ml) with or without IFN*α* (1,000 U/ml). For pre-IFN, IFN*α* was added 6 h before stimulation with anti-IgG/IgM antibody. After 2 d, the expression levels of FOXM1 and CCNA2 were quantitated *via* RT-PCR. The error bars represent SEM. Each expression level of FOXM1 and CCNA2 was standardized by each corresponding expression level of 18s rRNA, and the standardized expression levels of unstimulated B cells on day 0 were regarded as 1. The error bars represent SD. Data are representative of experiments from three independent donors. Statistical analysis was conducted *via* one-way ANOVA using Tukey’s multiple comparison test. *p < 0.05, **p < 0.01, and ***p < 0.001.

We assessed the effects of pre-IFN on B cell activation following stimulation with anti-IgG/IgM antibody *via* flow cytometry. Pre-IFN significantly enhanced cell enlargement (**Figure 3A**) and cell division (**Figure 3B**) following stimulation with anti-IgG/IgM antibody.

The interaction of CD40 on B cell and CD40 ligand (CD40L) on T cell is one of the main systems providing T cell help to B cell activation (Quezada, ARI, 2004). To examine the effect of pre-IFN on T cell help, CD40L was co-added in B cell culture. CD40L counteracted the effects of pre-IFN on cell enlargement and cell division stimulated by anti-IgG/IgM antibody (**Figures 3A, B**), although co-stimulation with anti-IgG/IgM antibody and CD40L also enhanced B cell activation, as determined *via* the evaluation of cell size and cell division. Pre-IFN improved cell survival in unstimulated and activated B cells (**Supplementary Figure 5**). Subsequently, we compared the gene induction of FOXM1 and CCNA2 (a downstream molecule of FOXM1) between cultures with and without pre-IFN after stimulation with anti-IgG/IgM antibody. The expressions of FOXM1 and CCNA2 were significantly increased in IFN*α* pretreated B cells more than those cultures without IFN*α* (**Figure 3C**).

### Pre-IFN Promoted Plasmablast Differentiation in B Cells Stimulated With Anti-IgG/IgM Antibody

To examine the effects of pre-IFN on plasmablast differentiation, the induction of CD38^+^CD43^+^ B cells after stimulation with anti-IgG/IgM was evaluated *via* flow cytometry. Plasmablast differentiation required pre-IFN in B cells stimulated by anti-IgG/IgM (**Supplementary Figure 6**).

### Pre-IFN Enhanced B cell Signaling After BCR Triggering

**Figure 4:**
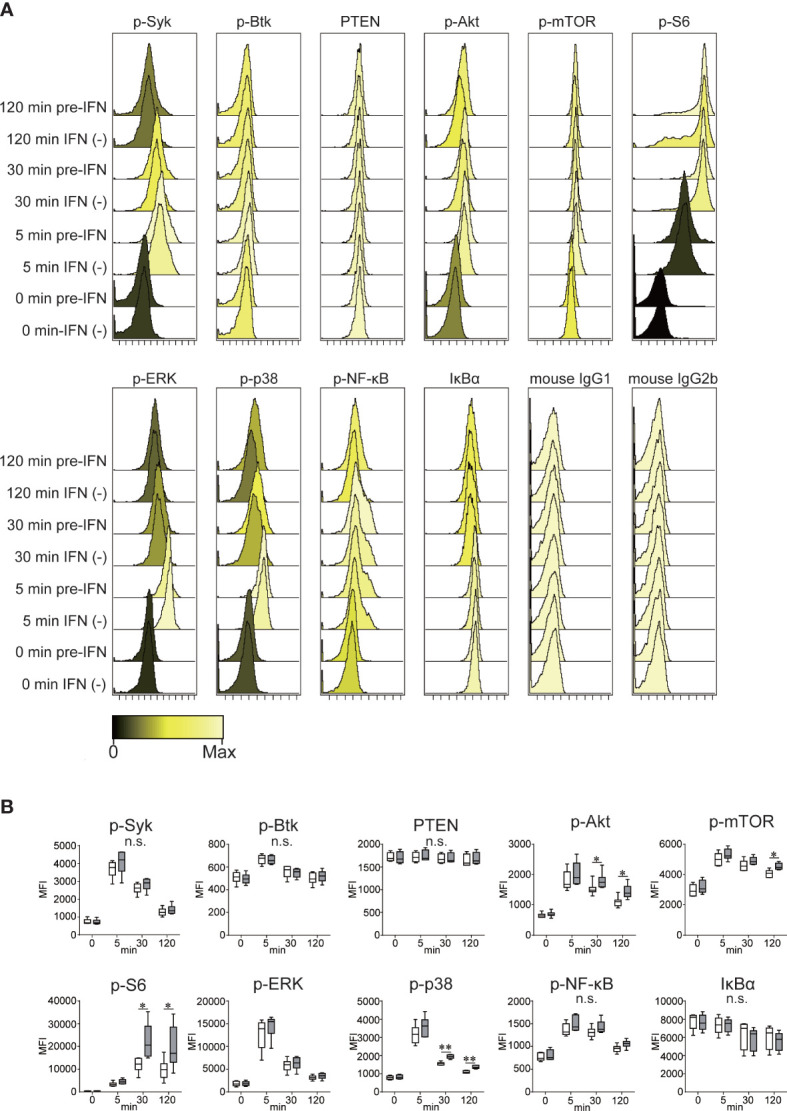
Enhanced BCR signaling pathways with pretreatment with IFN*α*. Isolated B cells from six healthy donors were stimulated with *α*-IgG/IgM with or without [IFN(−)] pre-IFN. Cells were harvested before stimulation (0 min) and 5, 30, and 120 min after stimulation with *α*-IgG/IgM antibody (50 µg/ml). After fixation and permeabilization, cells were stained with Alexa Fluor 647-labeled anti-PTEN, phosphoproteins (p-Syk, p-Btk, p-Akt, p-mTOR, p-S6, p-ERK, p-p38, and p-NF-κB), IkB*α*, mouse IgG1 isotype, and mouse IgG2b isotype. The expressions of these molecules were quantified *via* flow cytometry. **(A)** The histogram indicates the expression level of B cell signaling molecules at the indicated times after stimulation. Data in **(A)** are representative data from experiments of six independent donors. **(B)** Time kinetics of the expression levels of phosphorylated (except PTEN and IkB*α*) B cell signaling molecules after stimulation. Data of the mean fluorescence intensity (MFI) of each signaling molecule are presented as box plots (n = 6, each). Each box represents the 25^th^–75^th^ percentile. Lines inside the boxes indicate the median. Whiskers represent the 10^th^–90^th^ percentile. Comparisons between stimulation with (gray box) and without (white box) pre-IFN were statistically performed *via* an unpaired *t*-test. *p < 0.05, **p < 0.01, and n.s., not significant between IFN (−) and pre-IFN.

To determine what BCR (B cell receptor) signaling pathway was promoted by pre-IFN*α*, we examined the phosphorylation of BCR signaling proteins *via* flow cytometry. As presented in **Figure 4**, multiple proteins were prominently phosphorylated after BCR triggering with anti-IgG/IgM antibody. I*κ*B*α* was reciprocally degraded by NF-*κ*B phosphorylation, which indicated that stimulation with anti-IgG/IgM antibody also activated the NF-*κ*B pathway. Contrary to the pan-activation of B cell signaling pathways with BCR triggering (except PTEN), pre-IFN preferentially enhanced the phosphorylation of Akt, mTOR, S6, and p38 (**Figure 4B**). Western blotting also revealed that pre-IFN increased phosphorylation of Akt and p38 after stimulation anti-IgG/IgM antibody. Pre-IFN enhanced the phosphorylation of Akt and S6 after stimulation with anti-IgG/IgM antibody and CD40L (**Supplementary Figure 7**), as well as without CD40L (**Figure 4B**). In addition, co-stimulation with CD40L significantly promoted the activation of p38 and NF-*κ*B pathways stimulated with BCR, and the effects of pre-IFN on the activation of these pathways were blunted by co-stimulation with CD40L (**Supplementary Figure 8**).

### FDI-6 Inhibited B Cell Division and Enhanced Apoptotic B Cell Death in Stimulated and Pre-IFN-Treated B Cells

**Figure 5:**
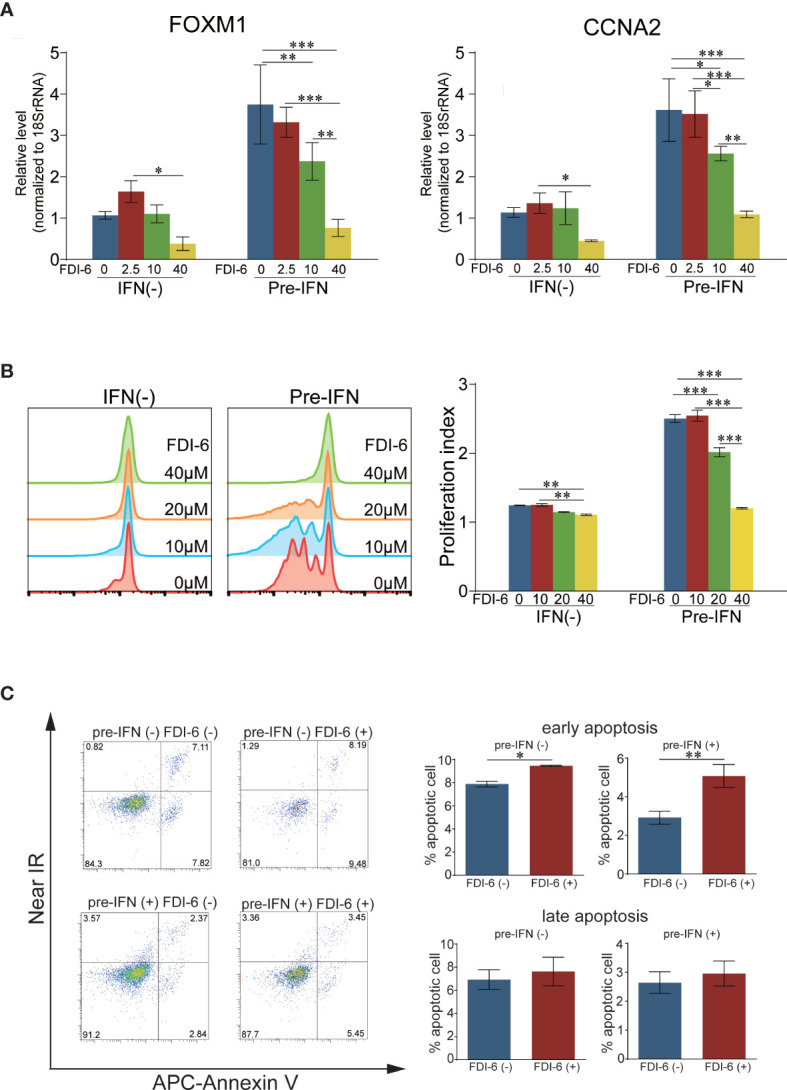
Effects of FDI-6 on cell division and apoptosis in activated B cells through BCR stimulation. **(A)** The effects of FDI-6 on FOXM1 and CCNA2 expression in stimulated B cells. Isolated B cells from a healthy donor were stimulated with *α*-IgG/IgM (50 µg/ml) with or without pre-IFN (1,000 U/ml). The addition of DMSO or FDI-6 (2.5, 10, or 40 µM) was performed 2 d after the start of the culture, and each cultured B cell was harvested 24 h (3 d after the start of the culture) after the addition of DMSO or FDI-6. The expressions of FOXM1 and CCNA2 were quantitated *via* RT-PCR. Each expression level of FOXM1 and CCNA2 was standardized by each corresponding expression level of 18s rRNA, and the standardized expression levels of non-IFN*α*-treated B cells without FDI-6 were regarded as 1. **(B)** The effects of FDI-6 on cell division of activated B cells. Isolated B cells from a healthy donor were labeled using CellTrace Violet Cell Proliferation Kit and stimulated with *α*-IgG/IgM (50 µg/ml) with or without pre-IFN (1,000 U/ml). DMSO or FDI-6 (10, 20, or 40 µM) was added 2 d after the start of the culture, and cell division was analyzed *via* flow cytometry 5 d after stimulation with anti-*α*-IgG/IgM. Cell division is presented as histograms (left). PI was calculated in each stimulated B cell. **(C)** Peripheral B cells from a healthy donor were stimulated with *α*-IgG/IgM (50 µg/ml) with or without pre-IFN (1,000 U/ml), and DMSO or FDI-6 (40 µM) was added 2 d after the start of the culture. In dot plots, cultured B cells were stained with APC-Annexin V and Near IR 24 h after the addition of DMSO or FDI-6, and cells were analyzed *via* flow cytometry (left). Annexin V^+^ Near IR^-^ B cells and Annexin V^+^ Near IR^+^ are defined as early and late apoptotic cells, respectively. The frequency of apoptotic B cells as a percentage of the overall B cells is presented in each cultured B cell (right). The error bars represent SD. Statistical analysis was conducted *via* one-way ANOVA using Tukey’s multiple comparison test **(B)** and student t-test **(C)**. *p < 0.05, **p < 0.01, and ***p < 0.001.

Microarray data indicated that FOXM1, and its downstream molecules were significantly upregulated in CD38^+^CD43^+^ B cells of SLE patients (**Figure 2**). We assumed that FOXM1 can be a candidate molecule for plasmablast-targeting therapy in SLE, as it is considered as a multipotent master transcriptional factor for cell proliferation and apoptosis (19). Then, we examined the effects of FOXM1 inhibitor FDI-6 on cell division and apoptosis in stimulated B cells with or without pre-IFN. FDI-6 significantly downregulated the expressions of FOXM1 and CCNA2 in B cells activated by anti-IgG/IgM and pre-IFN (**Figure 5A**). Cell division after stimulation with anti-IgG/IgM was significantly inhibited with FDI-6 and prominently with IFN*α* pretreatment (**Figure 5B**). Next, we evaluated the effects of pre-IFN on FDI-6-induced apoptosis in B cells activated by anti-IgG/IgM *via* flow cytometry. FDI-6 had just mild effect on induction on early apoptosis of activated B cells (**Figure 5C**).

## Discussion

Plasmablasts are the precursors of plasma cells in the peripheral blood, which produce autoantibodies in the bone marrow (28). Targeting plasmablasts may be an effective novel therapeutic strategy in the treatment of SLE, as plasmablasts increased in clinically active SLE patients and contained autoantibody-producing cells (25, 28, 29). In the present study, we investigated the gene expression of B cell subsets from SLE patients and healthy donors to elucidate the biological properties of plasmablasts of SLE patients. This may aid in the development of plasmablast-targeting therapy for SLE. Although the phenotype of plasmablasts in peripheral blood was defined as CD19^lo^CD27^hi^ (25), this population was overlapped with memory B cells (CD19^+^CD27^+^) and is difficult to be gated and sorted *via* flow cytometry. It has been reported that CD20^+^CD27^+^CD43^+^ B cells are capable of producing antibodies and cellular properties similar to plasmablasts (26, 30). We confirmed that CD38^+^CD43^+^B cells were highly overlapped with CD19^lo^CD27^hi^ B cells (**Figure 1A**) and clearly gated and sorted with high purity *via* flow cytometry (**Supplementary Figure 3**). We also demonstrated that isolated CD38^+^CD43^+^ B cells secreted IgG without any B cell stimulation (**Figure 1D**), which is one of the most important characteristics of plasmablasts (29). These findings support CD38^+^CD43^+^ as a cell surface phenotype of plasmablasts.

Few reports have identified the distinct molecular signature in isolated plasmablasts of active SLE patients compared with healthy controls, although several groups have reported that PBMC (2, 3) and B cell subsets (18, 31–34) in SLE patients express higher levels of ISGs. Lugar et al. previously characterized circulating SLE plasma cells (CD19^dim^ IgD^−^CD38^++^) as more similar to mature tonsil plasma cells (CD19+ IgD^−^CD38^++^) than to tonsil plasmablasts (CD19^+^ IgD^+^CD38^+++^). Cell-cycle-related genes were not reported to be upregulated in circulating plasma cells, probably due to the lack of comparison with the counterparts of healthy donors, although they reported increased levels of type I IFN gene signatures in all subsets of B cell (31). Our analysis of isolated B cell subsets from only four donors each cannot find the heterogeneity of the disease status of SLE and gene expression profiling of single cell level in each B cell subset. Banchereau et al. reported that the whole blood transcriptome of 158 pediatric SLE patients identified a plasmablast signature as the biomarker that most strongly correlated with the disease activity of SLE (33). However, the strong association of the plasmablast signature with the disease activity of SLE may represent the association between disease activity and circulating plasmablasts (29), as discussed by the authors (33). Recently, Nehar-Belaid reported that single-cell RNA sequencing identified seven B cell subclusters and two plasma cell subclusters in pediatric SLE patients (18). Although three B cell subclusters and one plasma cell subcluster were shown to express ISGs, the distinct expression of cell-cycle-related genes among the subclusters was not identified. This inconsistency with the results of the present study may be caused by the loss of highly proliferative plasmablasts in the cryopreservation and thawing processes, although it is reported that cryopreservation does not lose the antibody-secreting capacity of plasmablasts in PBMC (35, 36). The present study is the first to report that cell-cycle-related genes, FOXM1, and its downstream genes, as well as ISGs, were elevated to a higher degree in freshly isolated plasmablasts of SLE patients compared with those of healthy donors. This indicates that the plasmablasts of SLE patients are not only quantitatively but also qualitatively distinct from those of healthy donors. The detailed molecular mechanism for enhanced cell cycles remains unknown, although type I IFN has been considered to contribute to the pathogenesis of SLE in terms of B cell abnormality (37, 38). In the present study, the results of the microarray analysis suggested the possible causal relation between type I IFN signature of SLE naïve and memory B cell subsets and cell cycle signature in SLE plasmablasts (**Figure 2B**).

Higher ISG expression in all B cell subsets may have been polyclonally induced by bystander exposure with type I IFN secreted from the plasmacytoid DC (39, 40) and/or epigenetic change in type I IFN genes in SLE B cells (41). The high expression of ISG may be one of the “intrinsic” B cell characteristics of SLE before the disease onset, and we assumed that IFN signature in the B cells of SLE patients may be similar to that in B cells *in vitro* with pre-IFN. Hence, we designed the experiments as follows: first, B cells isolated from healthy donors were incubated with IFN*α* to induce ISGs, and cells were stimulated and analyzed to elucidate the cellular behavior of B cells with high ISG expression following B cell stimulation. BCR triggering induced the phosphorylation of kinases, such as Syk, leading to the activation of downstream PI3 kinase pathway (42), which activated the Akt–mTOR–S6 axis (43). Consistent with our results, previous reports revealed that IFN*α* enhanced stimulation *via* BCR triggering (44–47). Analysis of phosphoproteins *via* flow cytometry revealed that pre-IFN selectively enhanced signal transduction *via* the Akt–mTOR–S6 and p38 pathways rather than the pan-B cell signaling pathways. A previous study reported that IFN*α* promoted the survival of human primary B-lymphocytes *via* phosphatidylinositol 3-kinase (PI3K) (48), which selectively activated the B cell signaling pathways. In another previous study, tyrosine phosphorylation was enhanced after BCR stimulation in SLE patients compared with healthy donors (49), and hyperactivity of Akt in B cells was due to the enhanced tyrosine phosphatase (50), which may support our results. Although several studies have reported that the lower PTEN expression in B cells in SLE patients may cause hyperresponsiveness when stimulated with anti-IgM and IL-21 (51), the results of flow cytometry in the present study revealed that pre-IFN exhibited no effect on PTEN expression (**Figure 4**). The identification of a gene (or genes) among ISGs, which may be responsible for selectively enhancing BCR signaling, is very important. As such, further studies are required to elucidate the precise mechanism of the effects of pre-IFN on BCR signaling pathways. The ligand for CD40 (CD40L), which is expressed on follicular helper T cells (Tfh) and B cells, is considered to receive signals *via* CD40 from Tfh, mainly in the germinal centers (52). CD40 signaling is required for somatic hypermutation and humoral immune memory generation (53). CD40 ligation activates NF-κB, JNK, p38, and PI3 kinase *via* TRAF family proteins, depending on the cell type (53). During co-stimulation with CD40L, the enhancing effects of pre-IFN on BCR-induced blastic change (**Figure 3A**) and cell division (**Figure 3B**) were abolished. This may be due to the remarkable activation of NF-*k*B and p38. Moreover, only when the combination of BCR and CD40 triggering (**Supplementary Figure 8**) is so strong that the additional effect of pre-IFN seemed to be less clear. Although it was recently reported that anti-DNA autoreactivity was driven by type I IFN*α*, TLR7/9, and CD40–CD40L interaction in Dnase 1l3^−/−^ mice (54), our results revealed that pre-IFN granted proliferative and survival advantages to B cells stimulated *via* BCR triggering, even without CD40 ligation, which resulted in the activation of several B cells in SLE *via* extrafollicular pathways (55, 56) without the help of T cells.

FOXM1 is a transcriptional factor that regulates cell cycle and cell survival (19). Microarray analysis revealed that FOXM1 (**Figure 2C**) and its regulated genes (**Table 1**) were significantly increased in CD38^+^CD43^+^ B cells of SLE. This indicates that proliferation and survival in activated B cells and/or plasmablasts may be under the regulation of FOXM1. The Akt–FOXO3–FOXM1 axis is important in tumorigenesis (57). It is reported that p38 regulates E2F1, which was shown to be significantly upregulated in CD38^+^CD43^+^B cells (**Supplementary Figure 4**), and E2F1 directly induced FOXM1 (58). The increased induction of FOXM1 may have resulted from the convergence of these signaling pathways enhanced with pre-IFN, since Akt and p38 were preferentially enhanced with pre-IFN following BCR triggering (**Figure 4**). Buchner reported that FOXM1 inhibition exhibited a cytotoxic effect on B cells without any effect on normal B cell development and survival and suggested FOXM1 as a therapeutic target for acute lymphoblastic leukemia (59). Recently, arthritis-associated precursor macrophages (AToMs) containing osteoclast precursors were identified, and the differentiation of AToMs from osteoclasts was regulated by FOXM1. FOXM1 inhibitor inhibited osteoclastogenesis, indicating that it may be a potential target for the treatment of rheumatoid arthritis (60). FDI-6 is a direct inhibitor of the DNA binding domain of FOXM1 proteins and is a selective FOXM1 inhibitor that reduces the induction of downstream FOXM1 genes (61, 62). The promoter region of FOXM1 has FOXM1 binding sites (62), and FOXM1 itself induces its own transcription (63, 64). Consistent with these reports, FDI-6 reduced the upregulation of FOXM1 and CCNA2, especially in B cells with pre-IFN (**Figure 5A**). Our results in **Figure 5B** suggests that the specific inhibition of FOXM1 may have more anti-proliferative effect on SLE plasmablasts. Although the effect of FDI-6 on induction of apoptosis on activated B cell was very mild, the additional drug which inhibit compensatory signaling pathway may enhance the cytotoxic effect of FDI-6 as reported (65).

**Figure 6:**
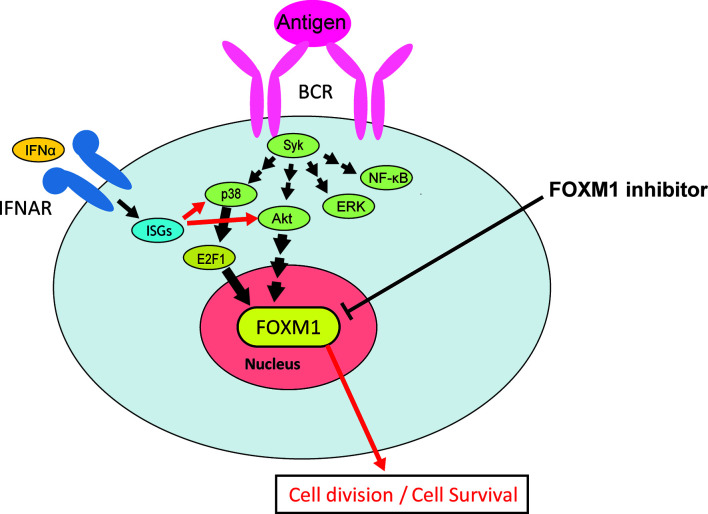
The effects of pretreatment of type I IFN on B cell stimulation, and the potential of FOXM1 inhibitor as a novel plasmablast-targeting therapy in SLE. Resting B cells (*e.g.*, naïve and memory B cells) were pretreated with type I IFN in an antigen non-specific manner, and ISGs were induced. After the stimulation of high-ISG-expressing B cells with BCR triggering, a particular gene (or genes) in the ISGs selectively enhanced the activation of the Akt and p38 pathways, which resulted in greater FOXM1 induction. FOXM1 inhibitor may have more anti-proliferative and cytotoxic effects on type I IFN pretreated activated B cells (*e.g.*, SLE plasmablasts).

In the present study, we revealed that pre-IFN upregulated numerous genes that were not ISGs after stimulation with BCR triggering. The type I IFN signatures were elevated more than 1 year prior to the disease onset (66), and type I IFN may play more significant roles in the early phase of the SLE pathology (15). The disease activity of SLE did not necessarily correlate with the IFN signatures (33). We should understand the distal effect of type I IFN exposure in considering the therapies targeting the whole type I IFN activated pathways, as the contribution of type I IFN to the pathogenesis in each SLE patient may not always be evaluated in terms of the expression levels of ISG. We proposed the model that pre-exposure of B cells to type I IFN polyclonally induces ISGs. BCR triggering of B cells with high levels of ISG activates Akt and p38 to a greater extent. The resulting elevated expression of FOXM1 and its regulated genes in plasmablasts in SLE may be the result of type I IFN exposure; hence, targeting this axis may be a novel strategy for the treatment of SLE (**Figure 6**).

## Data Availability Statement

The data sets presented in this study can be found in online repositories. The names of the repository/repositories and accession number(s) can be found in the article/**Supplementary Material**.

## Ethics Statement

The studies involving human participants were reviewed and approved by the Medical Ethical Review Board at Tohoku University School of Medicine. The patients/participants provided their written informed consent to participate in this study.

## Author Contributions

KA performed the experiments and wrote the manuscript, KY performed the experiments. TS, TI and HH discussed the data and critically revised the manuscript. HF designed and performed the experiments and wrote the manuscript. All authors contributed to the article and approved the submitted version.

## Funding

This work was supported by JSPS KAKENHI grant number 15K09515.

## Conflict of Interest

The authors declare that the research was conducted in the absence of any commercial or financial relationships that could be construed as a potential conflict of interest.
