# Supplementary figures and images for: Interferon α Enhances B Cell Activation Associated With FOXM1 Induction: Potential Novel Therapeutic Strategy for Targeting the Plasmablasts of Systemic Lupus Erythematosus
Source: Front Immunol. 2021 Feb 3;11:498703. doi: 10.3389/fimmu.2020.498703 (PMC7902015; doi:10.3389/fimmu.2020.498703)

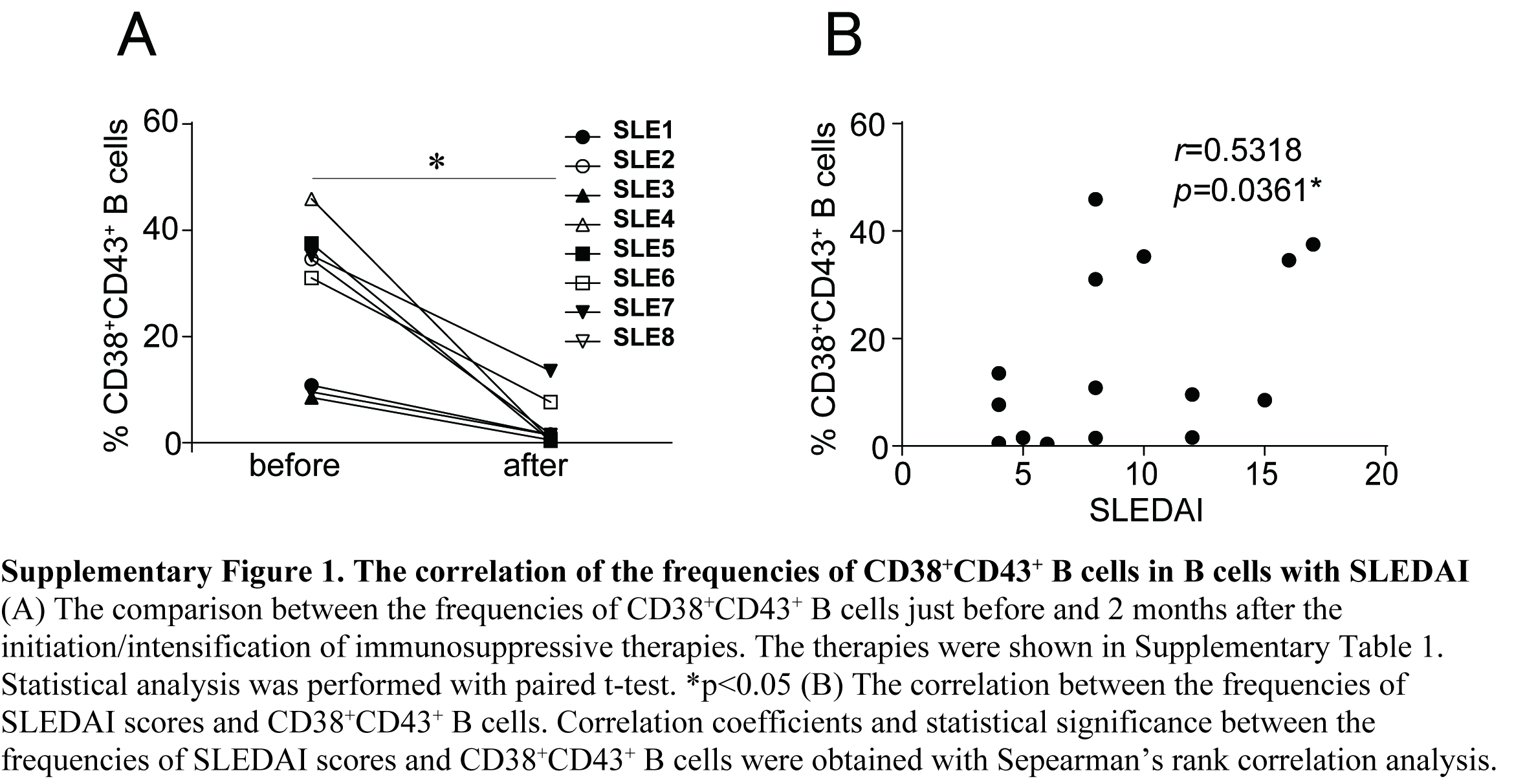

Supplement: Supplementary file 1 [file Image_1.tif]

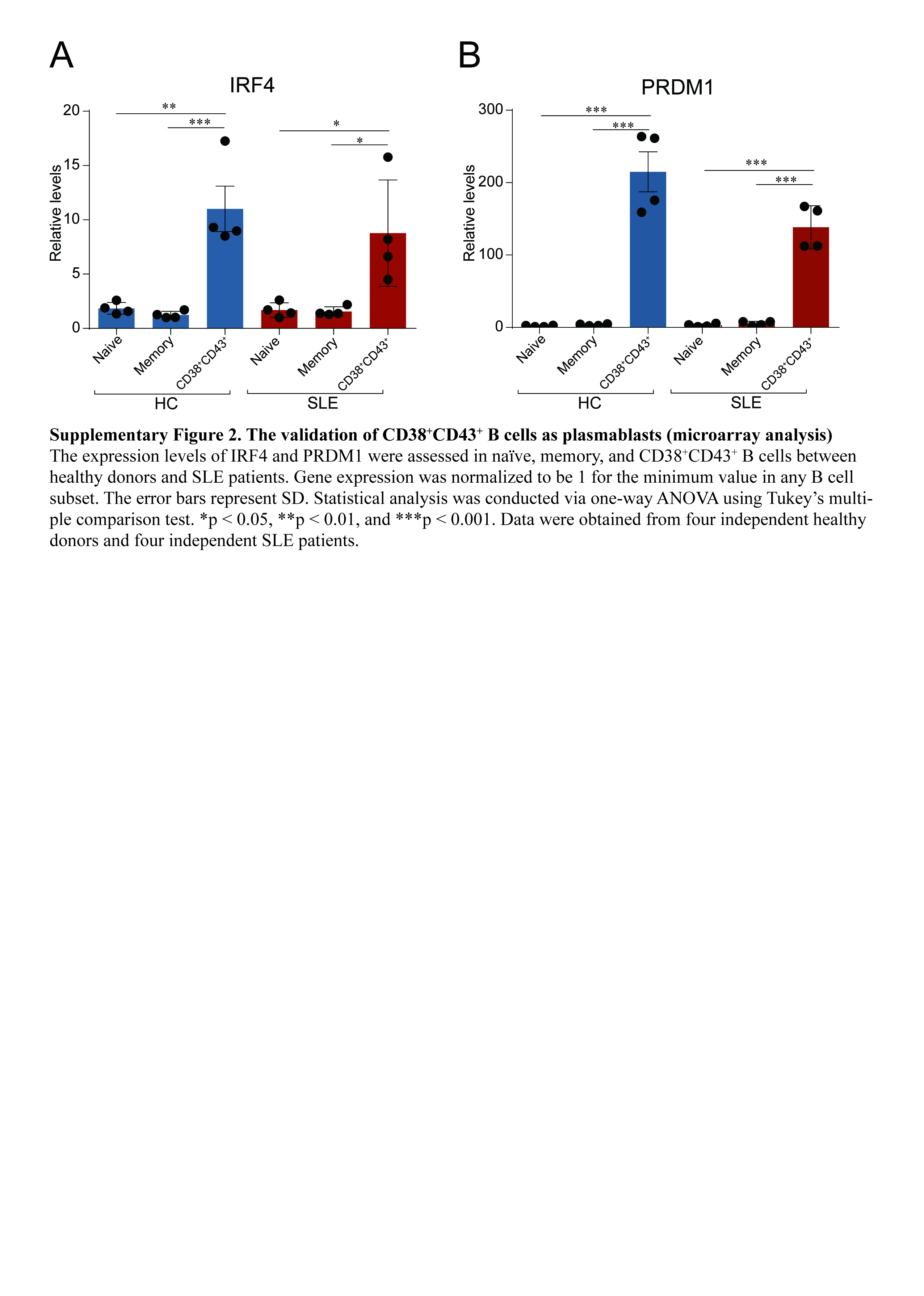

Supplement: Supplementary file 2 [file Image_2.tif]

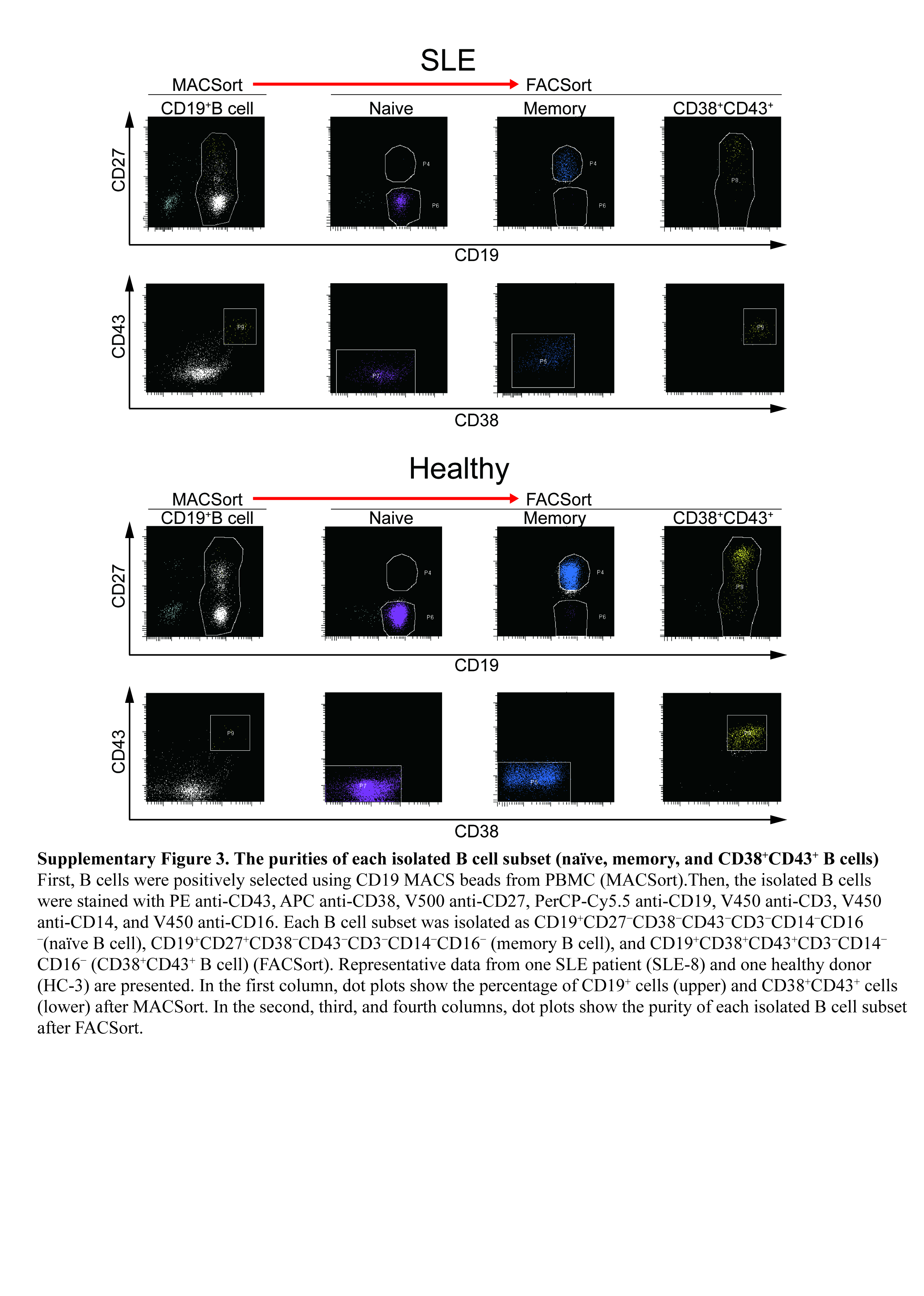

Supplement: Supplementary file 3 [file Image_3.tif]

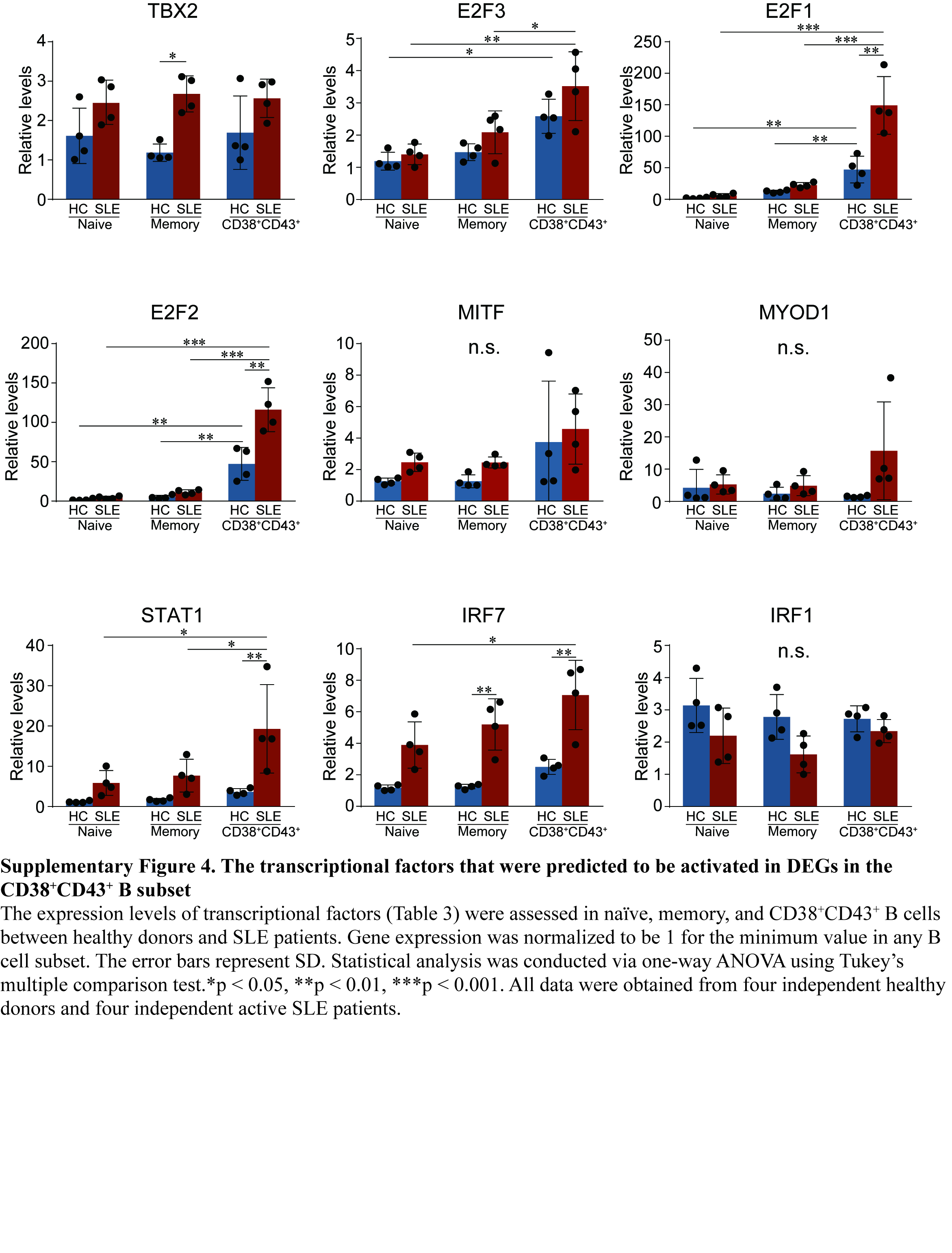

Supplement: Supplementary file 4 [file Image_4.tif]

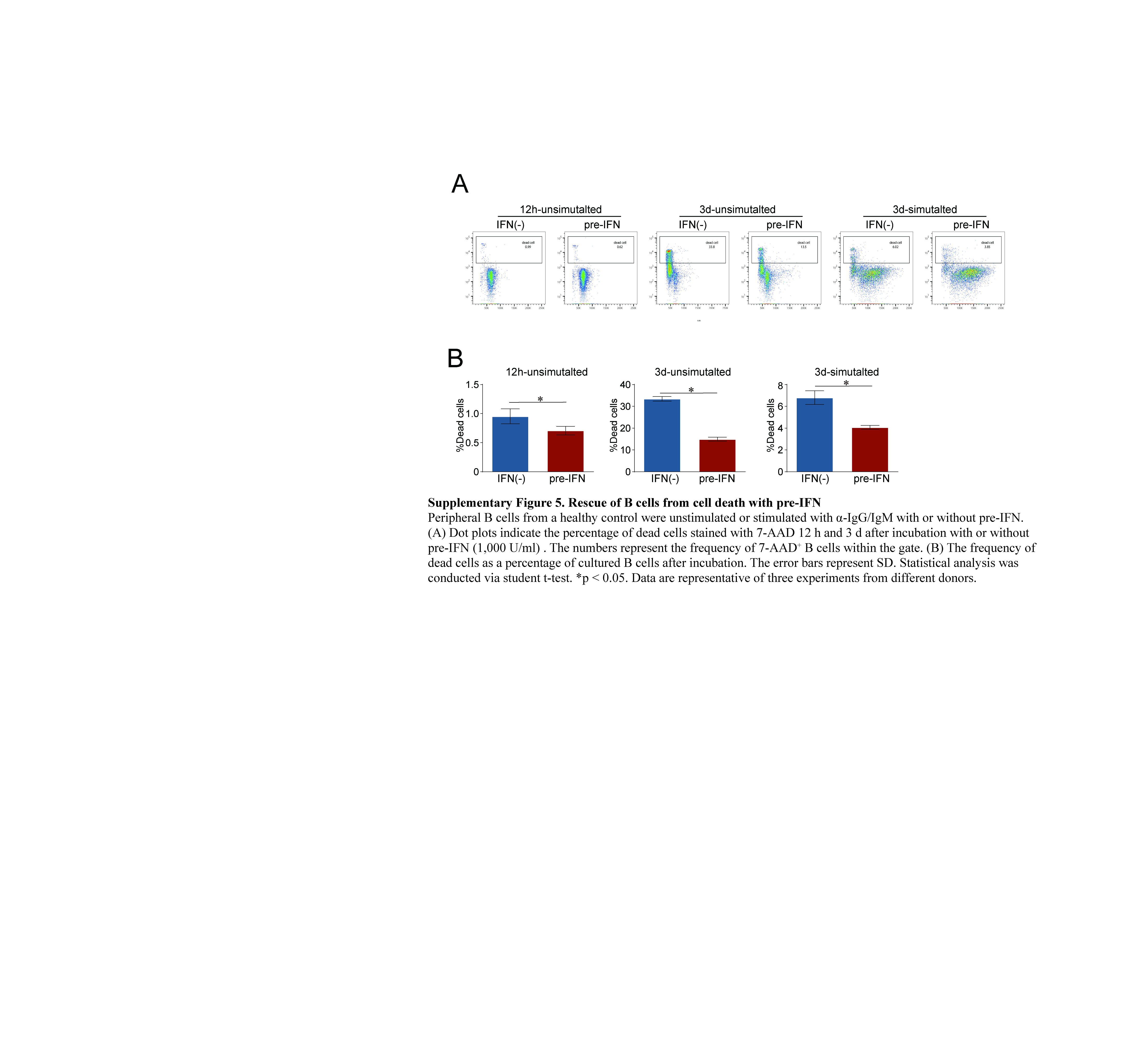

Supplement: Supplementary file 5 [file Image_5.tif]

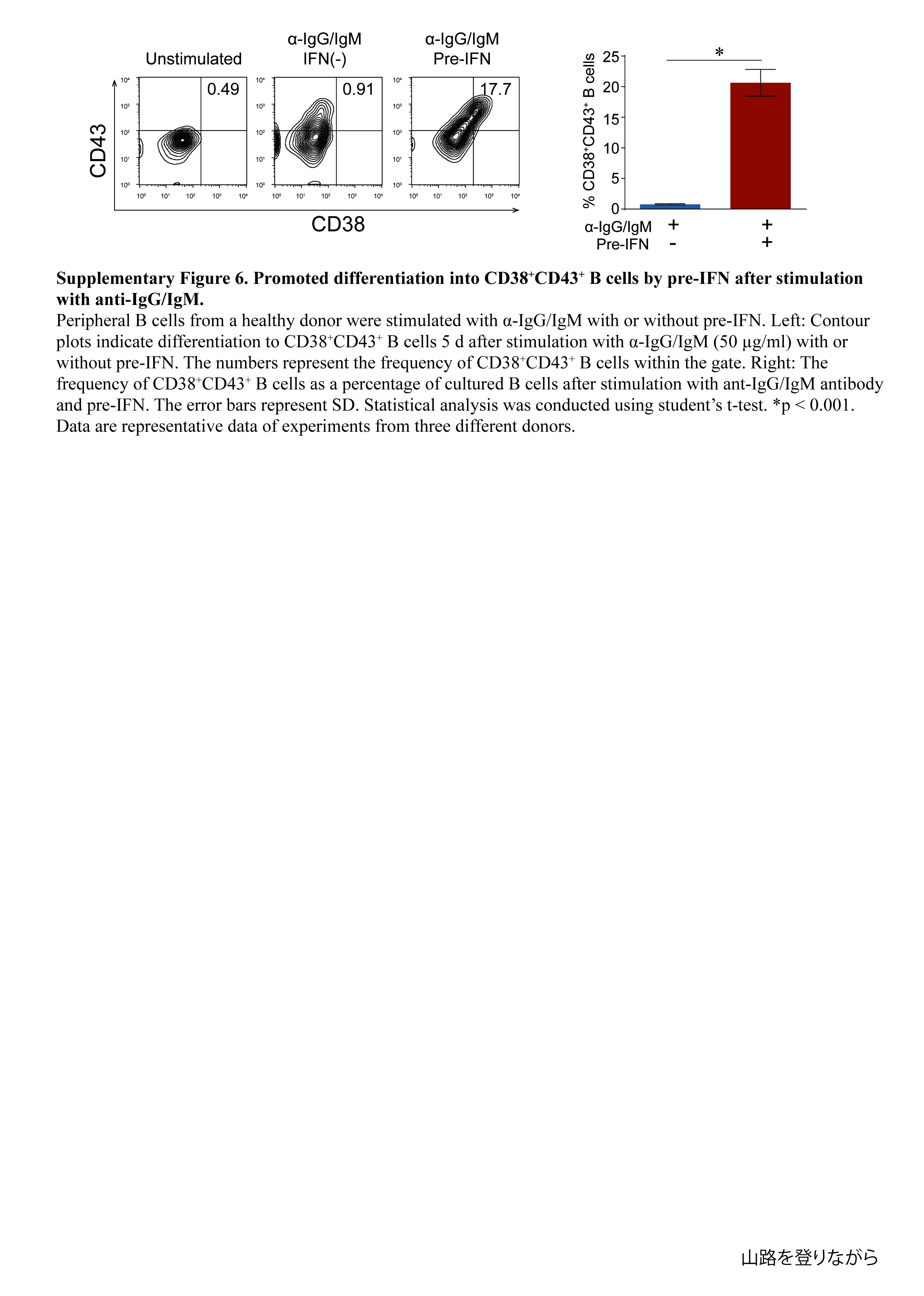

Supplement: Supplementary file 6 [file Image_6.tif]

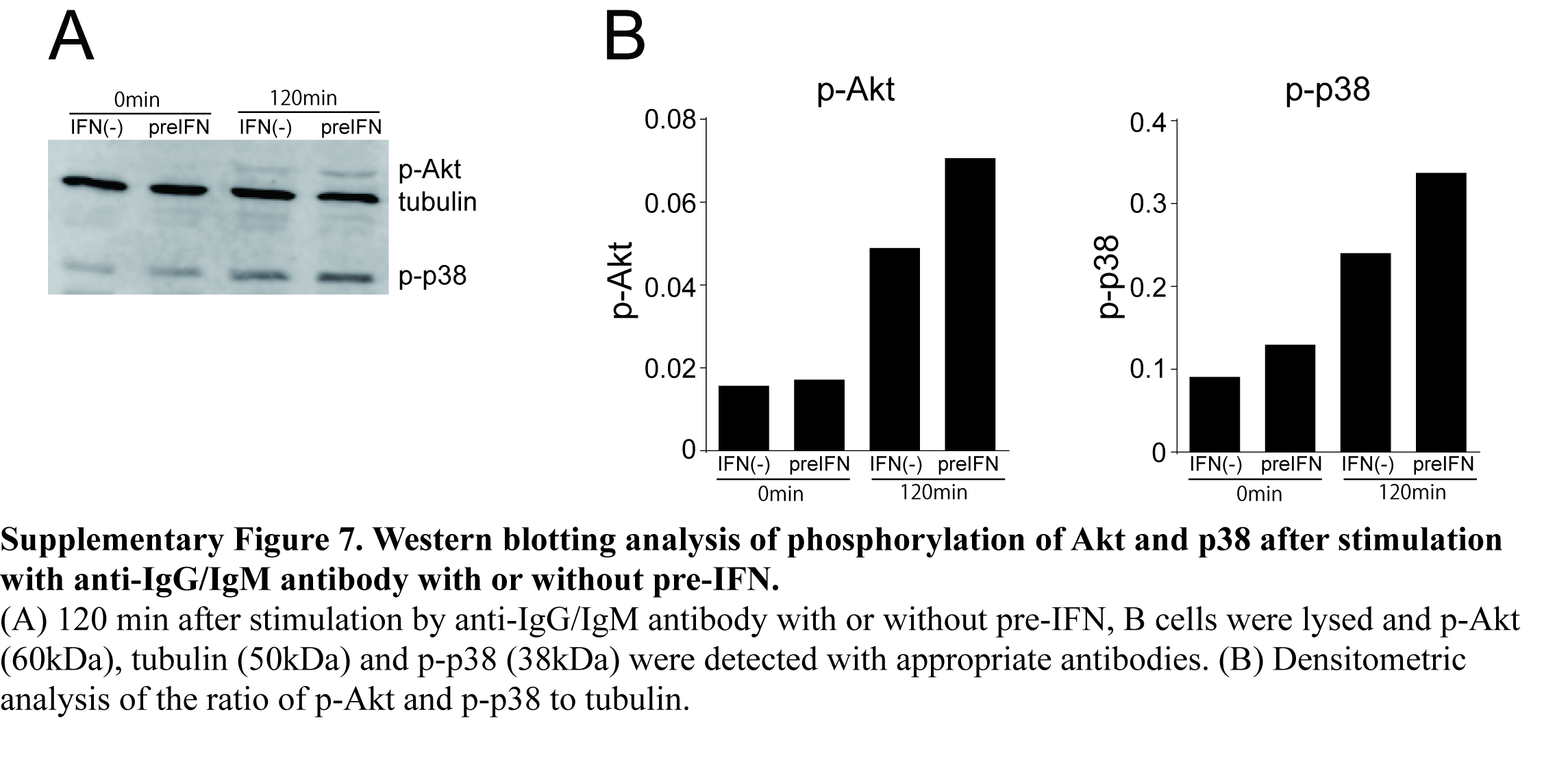

Supplement: Supplementary file 7 [file Image_7.tif]

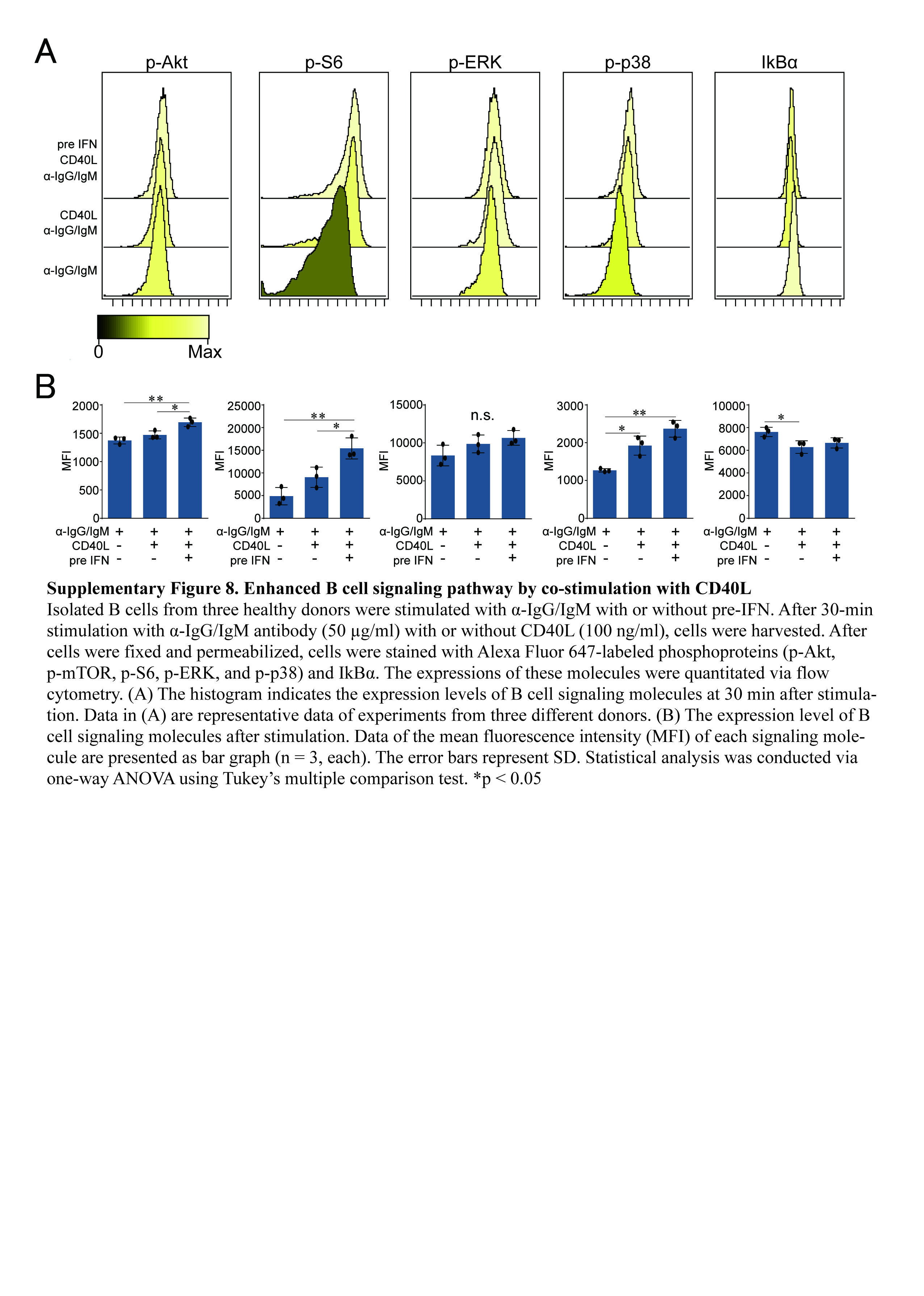

Supplement: Supplementary file 8 [file Image_8.tif]
