# Supplementary material for: Interferon α Enhances B Cell Activation Associated With FOXM1 Induction: Potential Novel Therapeutic Strategy for Targeting the Plasmablasts of Systemic Lupus Erythematosus
Source: Front Immunol. 2021 Feb 3;11:498703. doi: 10.3389/fimmu.2020.498703 (PMC7902015; doi:10.3389/fimmu.2020.498703)
Supplement: Supplementary file 9 [file Table_1.docx]

**Supplementary Table 1 Characteristics of SLE patients in clinical correlation**

|  | before | after |
| --- | --- | --- |
| SLE1 | untreated | IVMP, PSL60mg/day, IVCY |
| SLE2 | PSL 10mg/day | IVMP, PSL 60mg/day, IVCY |
| SLE3 | untreated | PSL 60mg/day, IVCY |
| SLE4 | PSL 20mg/day, MZB 150mg/day, AZA 150mg/day | IVMP, PSL 50mg/day, MTX 12mg/wk |
| SLE5 | untreated | IVMP, PSL50mg/day, IVCY |
| SLE6 | untreated | PSL 50mg/day |
| SLE7 | PSL 10mg/day, AZA 75mg/day | PSL 50mg/day, AZA 75mg/day |
| SLE8 | PSL 20mg/day, MZB 150mg/day | IVMP, PSL 60mg/day, IVCY, MZB 150mg/day |

AZA: azathioprine

IVCY: intravenous cyclophosphamide

IVMP: intravenous methylprednisolone (steroid pulse)

MTX: methotrexate

MZB: mizoribine

PSL: prednisolone
