# Supplementary material for: Interferon α Enhances B Cell Activation Associated With FOXM1 Induction: Potential Novel Therapeutic Strategy for Targeting the Plasmablasts of Systemic Lupus Erythematosus
Source: Front Immunol. 2021 Feb 3;11:498703. doi: 10.3389/fimmu.2020.498703 (PMC7902015; doi:10.3389/fimmu.2020.498703)
Supplement: Supplementary file 10 [file Table_2.docx]

**Supplementary Table 2** **Characteristics of SLE patients in microarray analysis**

|  | SLE profile | SLEDAI score |
| --- | --- | --- |
| SLE9 | ANA, anti-dsDNA Ab, nephritis, peritonitis,thrombocytopenia, PSL10mg, azathiopurine | 16 |
| SLE10 | ANA, anti-dsDNA Ab, face erythema, arthragia, nephritis, no treatment | 22 |
| SLE11 | ANA, anti-dsDNA Ab, arthralgia, pleuritis, no treatment | 10 |
| SLE12 | ANA, anti-dsDNA Ab, face erythema, nephritis, pancytopenia,  no treatment | 16 |
