# Supplementary material for: Interferon α Enhances B Cell Activation Associated With FOXM1 Induction: Potential Novel Therapeutic Strategy for Targeting the Plasmablasts of Systemic Lupus Erythematosus
Source: Front Immunol. 2021 Feb 3;11:498703. doi: 10.3389/fimmu.2020.498703 (PMC7902015; doi:10.3389/fimmu.2020.498703)
Supplement: Supplementary file 11 [file Table_3.docx]

**Supplementary Table 3 Top 30 of DEGs in each B cell subset**

**Upregulated genes in naïve B cell**

| Symbol | Entrez Gene Name | Fold Change |
| --- | --- | --- |
| IFI27 | interferon alpha inducible protein 27 | 76.92 |
| SPATS2L | spermatogenesis associated serine rich 2 like | 66.641 |
| LAG3 | lymphocyte activating 3 | 58.003 |
| CCR1 | C-C motif chemokine receptor 1 | 35.901 |
| LINC00487 | long intergenic non-protein coding RNA 487 | 35.07 |
| OASL | 2'-5'-oligoadenylate synthetase like | 34.209 |
| FCN1 | ficolin 1 | 31.613 |
| S100A12 | S100 calcium binding protein A12 | 28.944 |
| NOD2 | nucleotide binding oligomerization domain containing 2 | 26.624 |
| FGFBP2 | fibroblast growth factor binding protein 2 | 26.048 |
| IFI44L | interferon induced protein 44 like | 23.325 |
| HOXB6 | homeobox B6 | 21.246 |
| RSAD2 | radical S-adenosyl methionine domain containing 2 | 21.136 |
| IFIT3 | interferon induced protein with tetratricopeptide repeats 3 | 21.13 |
| CHST6 | carbohydrate sulfotransferase 6 | 20.944 |
| NKG7 | natural killer cell granule protein 7 | 20.361 |
| UCHL1 | ubiquitin C-terminal hydrolase L1 | 19.754 |
| ELOVL3 | ELOVL fatty acid elongase 3 | 19.619 |
| NRIR | negative regulator of interferon response | 19.534 |
| BFSP2 | beaded filament structural protein 2 | 18.406 |
| CMPK2 | cytidine/uridine monophosphate kinase 2 | 17.638 |
| IFIT1 | interferon induced protein with tetratricopeptide repeats 1 | 17.628 |
| DHRS9 | dehydrogenase/reductase 9 | 17.281 |
| PNMA2 | PNMA family member 2 | 16.25 |
| LGALS2 | galectin 2 | 15.626 |
| H2BU1 | H2B.U histone 1 | 15.343 |
| ZNF683 | zinc finger protein 683 | 15.298 |
| CST3 | cystatin C | 15.205 |
| BIK | BCL2 interacting killer | 14.977 |
| ACTA2 | actin alpha 2, smooth muscle | 14.763 |

**Downregulated genes in naïve B cell**

| Symbol | Entrez Gene Name | Fold Change |
| --- | --- | --- |
| LCN8 | lipocalin 8 | -27.372 |
| FBLN2 | fibulin 2 | -21.908 |
| CAMK2N1 | calcium/calmodulin dependent protein kinase II inhibitor 1 | -19.507 |
| CD6 | CD6 molecule | -18.492 |
| FAM239B | zinc finger protein 839 pseudogene | -17.744 |
| ARHGEF10L | Rho guanine nucleotide exchange factor 10 like | -17.278 |
| SLC38A11 | solute carrier family 38 member 11 | -9.281 |
| ZBTB16 | zinc finger and BTB domain containing 16 | -8.82 |
| ESR2 | estrogen receptor 2 | -8.56 |
| FOXP1 | forkhead box P1 | -8.543 |
| AUTS2 | activator of transcription and developmental regulator AUTS2 | -8.264 |
| TMEM71 | transmembrane protein 71 | -7.848 |
| LCN15 | lipocalin 15 | -7.457 |
| PRR20A | proline rich 20A | -7.386 |
| MEGF6 | multiple EGF like domains 6 | -6.935 |
| MID2 | midline 2 | -6.554 |
| ARRDC4 | arrestin domain containing 4 | -6.446 |
| PCBP3 | poly(rC) binding protein 3 | -6.41 |
| PTPRB | protein tyrosine phosphatase receptor type B | -6.376 |
| SETD7 | SET domain containing 7, histone lysine methyltransferase | -6.188 |
| ALKAL2 | ALK and LTK ligand 2 | -6.155 |
| SDK2 | sidekick cell adhesion molecule 2 | -6.128 |
| ARHGAP20 | Rho GTPase activating protein 20 | -6.117 |
| PXDNL | peroxidasin like | -5.932 |
| NEXMIF | neurite extension and migration factor | -5.659 |
| GYPE | glycophorin E (MNS blood group) | -5.652 |
| RHPN2 | rhophilin Rho GTPase binding protein 2 | -5.534 |
| MGP | matrix Gla protein | -5.421 |
| ACVR1C | activin A receptor type 1C | -5.412 |
| EML6 | EMAP like 6 | -5.357 |

**Upregulated genes in memory B cell**

| Symbol | Entrez Gene Name | Fold Change |
| --- | --- | --- |
| LAG3 | lymphocyte activating 3 | 114.948 |
| IFI27 | interferon alpha inducible protein 27 | 103.459 |
| SPATS2L | spermatogenesis associated serine rich 2 like | 66.503 |
| LINC00487 | long intergenic non-protein coding RNA 487 | 44.67 |
| FGFBP2 | fibroblast growth factor binding protein 2 | 38.322 |
| CHST6 | carbohydrate sulfotransferase 6 | 31.547 |
| TYMS | thymidylate synthetase | 25.895 |
| H3C15 | H3 clustered histone 15 | 23.13 |
| OASL | 2'-5'-oligoadenylate synthetase like | 21.618 |
| IFI44L | interferon induced protein 44 like | 21.364 |
| RSAD2 | radical S-adenosyl methionine domain containing 2 | 20.714 |
| PLAAT2 | phospholipase A and acyltransferase 2 | 16.943 |
| USP18 | ubiquitin specific peptidase 18 | 16.886 |
| PNMA2 | PNMA family member 2 | 16.535 |
| CMPK2 | cytidine/uridine monophosphate kinase 2 | 15.844 |
| USP41 | ubiquitin specific peptidase 41 | 14.962 |
| LOC105371215 | uncharacterized LOC105371215 | 14.61 |
| IFI44 | interferon induced protein 44 | 14.564 |
| IFIT1 | interferon induced protein with tetratricopeptide repeats 1 | 14.029 |
| CDT1 | chromatin licensing and DNA replication factor 1 | 13.186 |
| UBE2C | ubiquitin conjugating enzyme E2 C | 12.88 |
| MT1E | metallothionein 1E | 12.418 |
| CCR1 | C-C motif chemokine receptor 1 | 11.955 |
| BIK | BCL2 interacting killer | 11.884 |
| FADS1 | fatty acid desaturase 1 | 11.643 |
| GMPR | guanosine monophosphate reductase | 11.352 |
| CD2 | CD2 molecule | 11.249 |
| LINC00327 | long intergenic non-protein coding RNA 327 | 11.246 |
| CYMP | chymosin, pseudogene | 11.056 |
| AICDA | activation induced cytidine deaminase | 10.396 |

**Downregulated gene in memory B cell**

| Symbol | Entrez Gene Name | Fold Change |
| --- | --- | --- |
| LCN8 | lipocalin 8 | -27.372 |
| FBLN2 | fibulin 2 | -21.908 |
| CAMK2N1 | calcium/calmodulin dependent protein kinase II inhibitor 1 | -19.507 |
| CD6 | CD6 molecule | -18.492 |
| FAM239B | zinc finger protein 839 pseudogene | -17.744 |
| ARHGEF10L | Rho guanine nucleotide exchange factor 10 like | -17.278 |
| SLC38A11 | solute carrier family 38 member 11 | -9.281 |
| ZBTB16 | zinc finger and BTB domain containing 16 | -8.82 |
| ESR2 | estrogen receptor 2 | -8.56 |
| FOXP1 | forkhead box P1 | -8.543 |
| AUTS2 | activator of transcription and developmental regulator | -8.264 |
| TMEM71 | transmembrane protein 71 | -7.848 |
| LCN15 | lipocalin 15 | -7.457 |
| PRR20A | proline rich 20A | -7.386 |
| MEGF6 | multiple EGF like domains 6 | -6.935 |
| MID2 | midline 2 | -6.554 |
| ARRDC4 | arrestin domain containing 4 | -6.446 |
| PCBP3 | poly(rC) binding protein 3 | -6.41 |
| PTPRB | protein tyrosine phosphatase receptor type B | -6.376 |
| SETD7 | SET domain containing 7, histone lysine methyltransferase | -6.188 |
| ALKAL2 | ALK and LTK ligand 2 | -6.155 |
| SDK2 | sidekick cell adhesion molecule 2 | -6.128 |
| ARHGAP20 | Rho GTPase activating protein 20 | -6.117 |
| PXDNL | peroxidasin like | -5.932 |
| NEXMIF | neurite extension and migration factor | -5.659 |
| GYPE | glycophorin E (MNS blood group) | -5.652 |
| RHPN2 | rhophilin Rho GTPase binding protein 2 | -5.534 |
| MGP | matrix Gla protein | -5.421 |
| ACVR1C | activin A receptor type 1C | -5.412 |
| EML6 | EMAP like 6 | -5.357 |

**Upregulated genes in CD38^+^CD43^+^ B cells**

| Symbol | Entrez Gene Name | Fold Change |
| --- | --- | --- |
| IFI27 | interferon alpha inducible protein 27 | 231.039 |
| IFI44 | interferon induced protein 44 | 56.07 |
| IFI44L | interferon induced protein 44 like | 39.255 |
| USP41 | ubiquitin specific peptidase 41 | 25.468 |
| USP18 | ubiquitin specific peptidase 18 | 24.592 |
| RSAD2 | radical S-adenosyl methionine domain containing 2 | 24.315 |
| LAG3 | lymphocyte activating 3 | 19.74 |
| NRIR | negative regulator of interferon response | 16.808 |
| LINC00487 | long intergenic non-protein coding RNA 487 | 15.987 |
| CMPK2 | cytidine/uridine monophosphate kinase 2 | 13.324 |
| IFIT1 | interferon induced protein with tetratricopeptide repeats 1 | 12.793 |
| GMPR | guanosine monophosphate reductase | 12.479 |
| ADM | adrenomedullin | 12.209 |
| IFI6 | interferon alpha inducible protein 6 | 11.813 |
| OASL | 2'-5'-oligoadenylate synthetase like | 10.473 |
| MX1 | MX dynamin like GTPase 1 | 10.131 |
| LINC02817 | long intergenic non-protein coding RNA 2817 | 9.996 |
| BIRC5 | baculoviral IAP repeat containing 5 | 9.379 |
| IFITM1 | interferon induced transmembrane protein 1 | 9.267 |
| IFIT3 | interferon induced protein with tetratricopeptide repeats 3 | 9.077 |
| DLGAP2 | DLG associated protein 2 | 9.003 |
| GALNT18 | polypeptide N-acetylgalactosaminyltransferase 18 | 8.868 |
| H4C13 | H4 clustered histone 13 | 8.487 |
| SLC35D3 | solute carrier family 35 member D3 | 8.449 |
| H2BC7 | H2B clustered histone 7 | 8.314 |
| AP1S2 | adaptor related protein complex 1 subunit sigma 2 | 8.272 |
| XAF1 | XIAP associated factor 1 | 8.249 |
| LINC02831 | long intergenic non-protein coding RNA 2831 | 8.133 |
| FAM171A1 | family with sequence similarity 171 member A1 | 8.124 |
| H3C11 | H3 clustered histone 11 | 8.115 |

**Downregulated genes in CD38^+^CD43^+^ B cells**

| Symbol | Entrez Gene Name | Fold Change |
| --- | --- | --- |
| NIBAN3 | niban apoptosis regulator 3 | -24.241 |
| WFDC21P | WAP four-disulfide core domain 21, pseudogene | -24.159 |
| LCN8 | lipocalin 8 | -19.922 |
| MPEG1 | macrophage expressed 1 | -13.531 |
| P2RY14 | purinergic receptor P2Y14 | -13.485 |
| AFF3 | AF4/FMR2 family member 3 | -13.137 |
| LRRK2 | leucine rich repeat kinase 2 | -12.6 |
| KCNH8 | potassium voltage-gated channel subfamily H member 8 | -12.553 |
| COL19A1 | collagen type XIX alpha 1 chain | -12.529 |
| GPA33 | glycoprotein A33 | -12.517 |
| JAZF1 | JAZF zinc finger 1 | -12.126 |
| TRAF5 | TNF receptor associated factor 5 | -11.638 |
| MYO15B | myosin XVB | -11.606 |
| CD5 | CD5 molecule | -11.494 |
| SORL1 | sortilin related receptor 1 | -11.41 |
| PLEKHA1 | pleckstrin homology domain containing A1 | -11.346 |
| AUTS2 | activator of transcription and developmental regulator AUTS2 | -10.979 |
| PLEKHG1 | pleckstrin homology and RhoGEF domain containing G1 | -10.703 |
| MEGF6 | multiple EGF like domains 6 | -10.321 |
| MS4A7 | membrane spanning 4-domains A7 | -10.208 |
| FOXP1-IT1 | FOXP1 intronic transcript 1 | -9.893 |
| BACH2 | BTB domain and CNC homolog 2 | -9.859 |
| TMEM71 | transmembrane protein 71 | -9.848 |
| SIX1 | SIX homeobox 1 | -9.795 |
| FRZB | frizzled related protein | -9.557 |
| FAM239B | zinc finger protein 839 pseudogene | -9.447 |
| SMIM5 | small integral membrane protein 5 | -9.425 |
| LTB | lymphotoxin beta | -9.404 |
| CXCR5 | C-X-C motif chemokine receptor 5 | -9.308 |
| ABCB4 | ATP binding cassette subfamily B member 4 | -9.192 |
